# Characterization of polychaetes inhabiting estuaries and inner bays by composition analysis of amino acids and lactate enantiomers

**DOI:** 10.1038/s41598-024-55861-5

**Published:** 2024-03-06

**Authors:** Mayu Onozato, Wataru Shinohara, Yuichiro Osaka, Tatsuya Sakamoto, Maho Umino, Atsuko Nishigaki, Kenji Okoshi, Takeshi Fukushima

**Affiliations:** 1 Department of Analytical Chemistry, Faculty of Pharmaceutical Sciences, Toho University, 2-2-1 Miyama, Funabashi-shi, Chiba 274-8510 Japan; 2 Chiba Municipal Chiba High School, 9-46-1 Konakadai, Inage-ku, Chiba-shi, Chiba 263-0043 Japan; 3 Department of Environmental Science, Faculty of Science, Toho University, 2-2-1 Miyama, Funabashi-shi, Chiba 274-8510 Japan

**Keywords:** Environmental chemistry, Marine biology

## Abstract

In this study, we investigated the composition of free amino acids and lactate (Lac) in polychaetes in river estuaries and inner bays using chromatographic techniques. Both l-amino acids and d-amino acids (d-asparagine, d-alanine (d-Ala), d-serine, d-aspartic acid, and d-proline (d-Pro)) were detected, indicating that polychaetes contain some d-amino acids. Some polychaete species exhibited notable amino acid levels, such as glycine in Capitellidae sp. and *Thelepus* sp., d-Pro in *Glycera* sp., and β-Ala in *Scoletoma nipponica* and *Scoletoma* sp.. High d-Lac levels were detected in *Tylorrhynchus osawai* and *Hediste diadroma*, (691 and 797 μmol/100 g-wet, respectively), with the d-form exceeding 98%. *T. osawai* was dominant in the upper tidal-sensitive zone, wherein other organisms were less abundant because of low salinity (3–8 PSU). Seasonal differences in the concentrations of components in *T. osawai* were observed, particularly a significant increase in d-Lac in the reproductive period. Notably, the d-Lac concentrations of *T. osawai* were higher upstream than downstream. Thus, d-Lac might be involved in strategies underlying adaptations to low salinity and reproductive activity. These results suggest that both the d-form of Lac and amino acids may play certain physiological roles in the life of polychaetes.

## Introduction

Amino acids are crucial for the sustenance of aquatic invertebrates. While l-amino acids are constituents of proteins, d-amino acids, mainly d-alanine (d-Ala), is involved in physiological functions such as osmoregulation in bivalves^1^ and crustaceans^2–4^, oogenesis in sea urchins^5,6^, molting of the Kuruma prawn^7,8^, and induction of tolerance to hypoxia in bivalves^9^. Among them, as osmoregulation is critical in aquatic invertebrates living in estuarine areas with highly variable salinity, d-Ala storage in the body is indispensable for their life.

Polychaetes are invertebrates inhabiting various locations, including marine, brackish, and freshwater environments and coastal sediments, estuaries, and tidal flats. Polychaetes are a dominant group of benthic organisms^10,11^. However, the biological mechanisms underlying the life cycle events of polychaetes, such as spawning, growth, and adaptation to a rapidly changing environment remain unknown. Data on the concentration and availability of d-amino acids and l-amino acids in polychaetes are scarce. Similar to other aquatic invertebrates, free d-amino acids may play important physiological and ecological roles in polychaetes inhabiting various environments.

Anaerobic environments are often formed in estuaries and inner bays^12^. l-Lactate (Lac) is the end product of anaerobic metabolism. Because polychaetes live at the bottom of sediments, which are highly anaerobic, l-Lac accumulates in polychaetes. Additionally, because the opposite enantiomer, d-Lac, is found in mammals and in the marine organism, octopus^13,14^, we speculated that both l-Lac and d-Lac are found in polychaetes, and their contents and the ratio may be altered ecologically by habitat and/or seasonal differences.

Thus, the purpose of this study was to characterize the concentration of amino acids and Lac in polychaete species living in different habitats, including several sites in Tokyo Bay, where some major rivers flow in, and Mangoku-ura Lagoon, a bag-shaped inner bay where no major river flows in. The obtained data can indicate the characteristics of d- and l-amino acids and Lac included in coastal polychaetes across various species and habitats. The findings are expected to assist in clarifying unexplained mechanisms underlying spawning, growth, and adaptation to a rapidly changing environment in polychaetes in future.

Considering the above background, we first investigated the presence and concentration of free d- and l-amino acids and -Lac in polychaetes inhabiting estuaries and inner bays, where highly environmental changes occur, by using high-performance liquid chromatography (HPLC) connected with mass-spectrometry or fluorescence detection.

Cluster and similarity of percentages (SIMPER) analysis were conducted to evaluate the similarity of concentrations and composition of these components among the polychaetes. By comparing the levels of free d- and l-amino acid and -Lac among 10 species of polychaetes, we found that *Tylorrhynchus osawai* had a d-Lac concentration approximately 10–100 times higher than that of other polychaetes. Therefore, we focused on *T. osawai* and conducted two subsequent experiments. First, we collected them “monthly for 5 months” from the Arakawa River that flows into Tokyo Bay and measured free d- and l-amino acid and -Lac concentrations. A hierarchical clustering heatmap analysis was conducted to identify components showing significant seasonal changes. Second, we collected them “at two points with different salinity levels” from the Arakawa River and, consequently, found that several components were significantly altered depending on the salinity of the habitat in *T. osawai* for the first time.

## Methods

### Collection and preparation of polychaete homogenates

No special permits were required to perform field research because the study sites were not privately owned or legislatively protected. Protected and/or endangered species were not included in this study.

**Figure 1:**
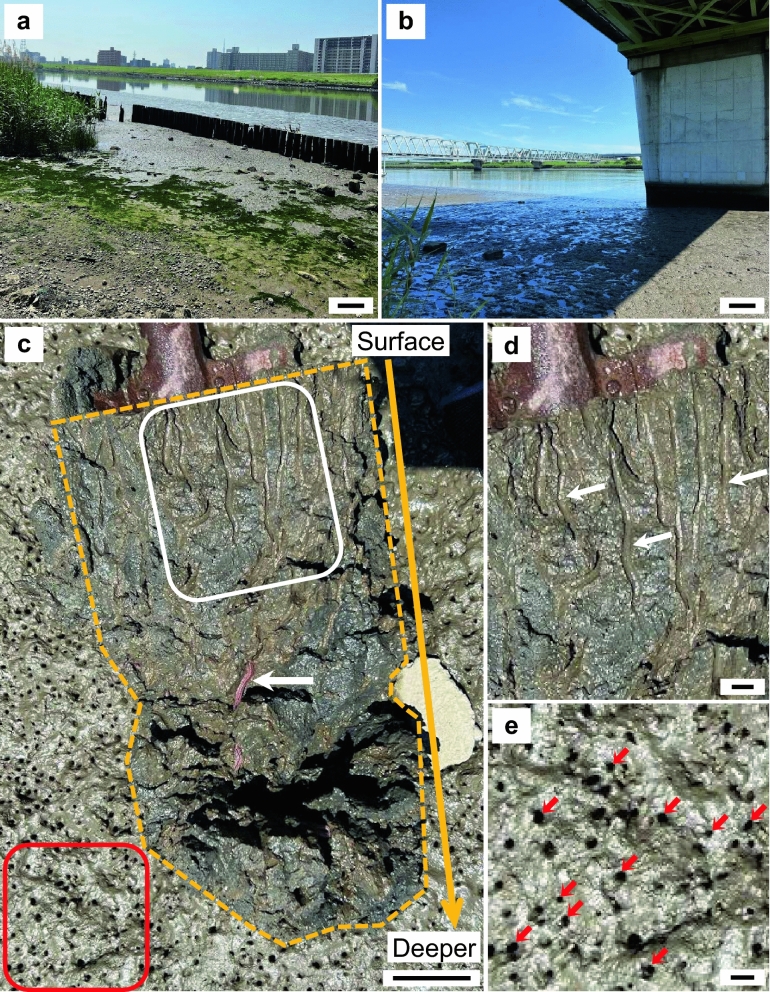
Collection sites of *T. osawai* at the Arakawa River. (**a**) Sampling site in the upstream region (scale bar: 1 m); (**b**) sampling site in the downstream region (scale bar: 1 m); (**c**) surface of the sediment and fracture surfaces of burrows of *T. osawai* (white square). The dug-up sediment is surrounded by a dashed yellow line, and the burrow is inhabited by *T. osawai* (white arrows). Numerous holes were observed in the surface sediment (red square, scale bar: 5 cm); (**d**) a magnified view of the region enclosed by the white frame. The white arrows indicate burrows, and the inner regions of the burrows (slightly brown zones) were more oxidative than the surrounding black zone (scale bar: 1 cm); (**e**) a magnified view of the holes on the sediment surface (red arrows, scale bar: 1 cm).

Ten species of polychaetes were collected at low tide from the intertidal zone by digging into the sediment or mud using a shovel between October 2021 and May 2023 (Fig. 1, Supplementary Fig. S1, and Supplementary Tables S1 and S2). *Marphysa* sp. E (Eunicidae)^15^ and *Arenicola brasiliensis* (Arenicolidae) were collected from the tidal flats of Tokyo Bay, our main study sites. *Hediste diadroma* (Nereididae) and *T. osawai* (Nereididae) were collected from the estuary of the Tamagawa River and the tidal reach of the Arakawa River, respectively, both of which flow into the Tokyo Bay. Among the polychaetes examined in this study, *T. osawai* inhabited the most upstream areas of the tidal-sensitive zone. It is the most dominant polychaete species in these areas. We focused on *T. osawai* to obtain novel insights into the biochemical properties of polychaetes because they may possess mechanisms of tolerance toward low salinity, which may be related to osmolytes, as they dominate at 10% of the salinity of open seawater (3 PSU). To examine the effects of salinity on the component concentrations and composition of *T. osawai*, we collected polychaetes from two sites of the Arakawa River (upstream: approximately 16 km from the river mouth, 3 PSU; downstream: 8 km from the mouth, 8 PSU). *T. osawai* was collected continuously between January and May 2023 from upstream areas to examine the concentration changes from winter to spring to clarify whether the component concentrations and composition of *T. osawai* are constant or affected by their life cycles (such as growth and reproductive periods) and the season (such as high or low temperatures). *Glycera* sp. (Glyceridae), *Marphysa* sp. A (Eunicidae)^15^, *Scoletoma nipponica* (Lumbrineridae), *Scoletoma* sp. (Lumbrineridae), Capitellidae sp. (Capitellidae), and *Thelepus* sp. (Terebellidae) were mainly collected from the artificial tidal flat and around Setojima Island in the Mangoku-ura Lagoon, northern Japan. Mangoku-ura Lagoon is a bag-shaped inner bay, where the intertidal zone was lost owing to the land subsidence of approximately 80 cm caused during the Great East Japan Earthquake in 2011^16,17^. Therefore, an artificial tidal flat (4 ha) was created at the mouth of the bay in 2016 for clam fishing. Mountain sand mixed with oyster shells was introduced into the tidal flat. The substrate of artificial tidal flats tends to be sandy mud mixed with oysters and clam shells. Setojima Island is located in the inner region of the bay with bottom sediment comprising soft mud. Various organisms, including polychaetes such as *Marphysa* sp, an asari clam *Ruditapes philippinarum*, and the Japanese mud shrimp *Upogebia major*^18^ inhabit these sites. Although the collection sites along the coast of Tokyo Bay are located around rivers and estuaries, the artificial tidal flat and Setojima Island is located on the Mangoku-ura Lagoon. The Mangoku-ura Lagoon is connected to the open sea by a narrow channel. The artificial tidal flat and Setojima Island are characterized by the absence of a major river inflow.

All polychaetes collected in this study were identifiable at the species level by morphological observations. Incomplete organisms (with bodies that were cut off at the time of collection and lacking heads or tails and only short fragments of which were available) and complete organisms that were challenging to identify were classified as belonging to the higher taxa to the extent that they could be identified.

Fresh living organisms were placed in plastic tubes filled with water collected at the collection sites and transported to the laboratory under refrigeration. For 1–2 d, the collected polychaetes were left in the local water and allowed to swim freely. Subsequently, they were stored at − 80 ℃ after fecal excretion. A piece of approximately 50–100 mg (excluding the head and feces) was cut from a thawed polychaete, placed into a 1.5-mL tube, and weighed. Ice-cold phosphate-buffered saline (PBS, 1.0 mL/100 mg sample) was added, and the sample was minced using a hand homogenizer and centrifuged at 13,200×*g* for 15 min at 4 ℃. The obtained supernatant was transferred to another 1.5-mL tube and diluted fivefold using PBS. The diluted supernatant (10 µL) was mixed with an organic solvent [CH_3_CN/CH_3_OH (1:1, *v*/*v*), 130 µL] in a 1.5-mL tube to prevent enzymatic reactions, vortexed for 1 min, and stored at − 80 ℃. Several samples were prepared in the same manner.

### Biomass estimation at the Arakawa River

Biomass estimation was employed to investigate the relationships between the salinities and abundances of *T. osawai* at two sites in the Arakawa River with different salinities. Quadrats (dimension: 25 × 25 cm) were placed at three or four arbitrary points within each study area (approximately 10 × 50 m) up- and downstream in the Arakawa River and photographs were taken from just above each quadrat. The sediment in each quadrat was dug up and sieved. Only *T. osawai* was collected and counted. Five or six organisms from each quadrat were placed into plastic tubes identical to those described above and transported to the laboratory. Each organism was weighed, and the mean ± standard deviation (SD) was calculated. The number of holes inhabited by *T. osawai* in each quadrat was counted using the photographs. Based on the number of holes and average weight, the biomass of *T. osawai* per square meter was calculated.

### Amino acid and Lac analysis

The reagents used to prepare and measure the samples are described in the Supplementary Information. The derivatization reagent used in amino acid analysis, (*R*)-CIMa-OSu, was synthesized in our laboratory as previously reported^19^.

In amino acid analysis, the frozen sample (diluted supernatant mixed with an organic solvent; kindly refer to the section *Collection and preparation of polychaete homogenates*) was thawed, derivatized with (*R*)-CIMa-OSu, purified using solid-phase extraction (SPE), and determined by using LC–MS/MS with minor modifications^20^.

In d- and l-Lac analysis, the thawed sample was fluorescence-derivatized with 4-nitro-7-piperazino-2,1,3-benzoxadiazole (NBD-PZ), subjected to SPE, and analyzed using column-switching HPLC-fluorescence detection^21,22^. A detailed explanation of the procedure is described in the Supplementary Information.

For analyzing an individual polychaete, samples were prepared in duplicates and each final solution was analyzed, with the mean taken as the component concentration of the polychaete. The concentrations of amino acids and Lac in each polychaete species are expressed as mean ± SD in µmol/100 g-wet.

### Statistical analysis

Cluster analysis was conducted based on the concentrations of amino acids and Lac detected in the extracts of 78 individuals belonging to 10 species to investigate the biochemical characteristics of each polychaete species. The Bray–Curtis similarity index^23^ was calculated based on the concentrations logarithmically transformed using the natural logarithm functions (ln(*x* + 1)) of the components. We performed cluster analysis using the group average method. The significance of the differences between groups in the cluster analysis was evaluated using permutational multivariate analysis of variance^24^. Even if only one individual of a species was collected, or if a species was not collected continuously across seasons, it was still included in the cluster analysis. SIMPER analysis was also performed to evaluate the components that contributed to the separation of the groups by comparing the separated groups based on their similarity.

To investigate the monthly variation in the constituents of *T. osawai*, a hierarchical clustering heatmap analysis was conducted using the concentrations of amino acids and Lac detected in *T. osawai* collected in the upstream region of the Arakawa River between January and May 2023. If the peaks of the target compounds were not determined (N.D.), zero was substituted with the limit of detection. One of the three organisms collected in January (as marked with * in Fig. 4) exhibited low similarity to the other two organisms in the cluster analysis described above. Thus, the means of each target compound were obtained for two individuals. The concentration of each component of *T. osawai* collected from February to May was expressed as a relative value, with the mean concentration of each component of *T. osawai* collected in January (*n* = 2) defined as 1.0. Subsequently, these relative values were transformed logarithmically (ln(*x* + 1)) and used to calculate the Bray–Curtis similarity index^23^. Next, we performed a hierarchical clustering heatmap analysis using the group average method. These analysis were performed using the R software (version 4.2.1, The R Foundation, Vienna, Austria).

We used the Mann–Whitney *U* test to compare the concentrations of components between the collection sites of *T. osawai*, the up- and downstream areas. One-way analysis of variance, followed by the Bonferroni test, was used to compare the concentrations of the components based on the months in which *T. osawai* was collected. A *P-*value of < 0.05 indicated statistical significance. These two comparisons were performed using BellCurve for Excel (Social Survey Research Information, Tokyo, Japan). Bar graphs with dot plots were created using Prism 8 (GraphPad Software, Boston, MA, USA).

## Results

### Chromatograms and mass spectra of the components of polychaete extracts

**Figure 2:**
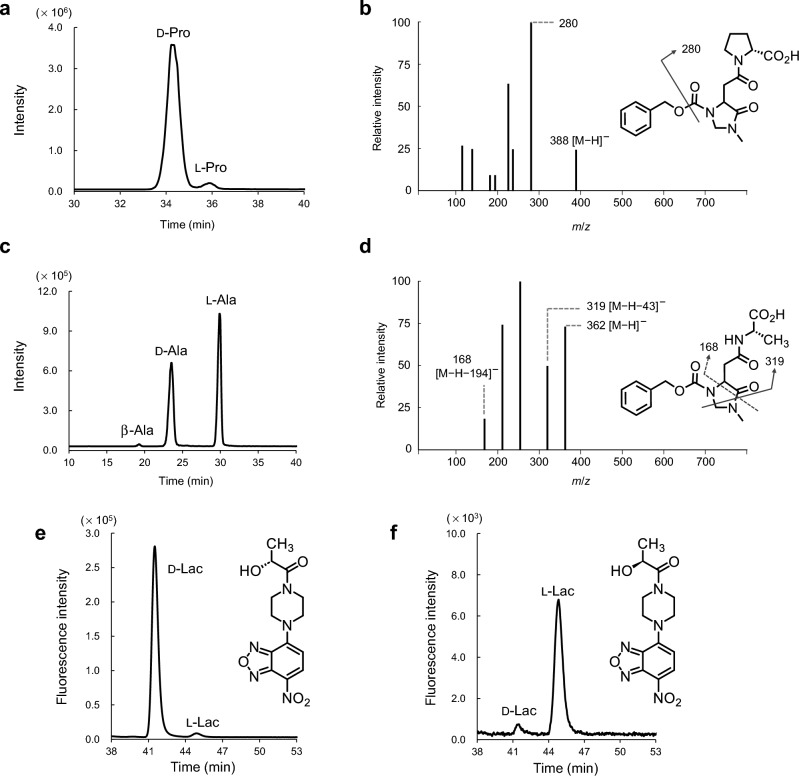
Chromatograms and CID mass spectra. (**a**) Chromatogram of d- and l-Pro within the *Glycera* sp. extract; (**b**) negative CID mass spectrum of the CIMa derivative of d-Pro and the fragmentation pattern; (**c**) chromatogram of β-, d-, and l-Ala in the *T. osawai* extract; and (**d**) negative CID mass spectrum of the CIMa derivative of d-Ala and the fragmentation pattern. Chromatograms of d- and l-Lac within the (**e**) *T. osawai* extract and the derivatized structure of d-Lac and (**f**) *Scoletoma* sp. extract and the derivatized structure of l-Lac.

Primary amino acids may be differentiated from secondary amino acids based on the fragmentation patterns of the mass spectra obtained by utilizing CIMa-OSu as a derivatization reagent^19^. Five d-amino acids (d-Asn, d-Ala, d-Ser, d-Asp, and d-Pro) were remarkably detected in polychaete extracts. A clear peak representing d-Pro was detected in the chromatogram of the *Glycera* sp. extract (Fig. 2a). The unique fragmentation pattern (de-PhCH_2_O product, *m*/*z* = 280) of CIMa-OSu coupled with Pro was obtained in the negative ion mode in collision-induced dissociation (CID) mass spectra (Fig. 2b). The fragment [M-H-43]^−^ was not observed in the CID mass spectrum of d-Pro derived from *Glycera* sp. (Fig. 2b). These mass spectral data indicate the presence of a secondary amino acid (Fig. 2b)^19^. Extremely large peaks representing d-Ala and β-Ala were detected in the chromatograms of the extracts of *T. osawai* (Fig. 2c) and *Scoletoma* sp. (Supplementary Fig. S2a), respectively. Two marked fragments, [M-H-43]^−^ (*m*/*z* = 319) and [M-H-194]^−^ (de-CbzNHCH_2_NHCH_3_ fragment, *m*/*z* = 168), were detected in the negative ion mode (Fig. 2d and Supplementary Fig. S2b). These spectral data (Fig. 2d and Supplementary Fig. S2b) indicate the presence of Ala, which is a primary amino acid^19^.

d,l-Lac in the polychaete extract was fluorescence-derivatized with NBD-PZ and detected using an octadecyl silica column (Supplementary Fig. S3a). d-Lac and l-Lac could be sufficiently separated through column-switching HPLC-fluorescence detection using a chiral column (Supplementary Fig. S3b). In the chromatogram of the *T. osawai* extract, the peak representing d-Lac was considerably larger than that representing l-Lac (Fig. 2e). The peaks representing d-Lac and l-Lac were detected in the chromatogram of the *Scoletoma* sp. extract (Fig. 2f). They were considerably smaller than those detected in the extract of *T. osawai*. Notably, the l-Lac peak was larger than the d-Lac peak.

### Concentrations and ratios of the components of polychaete extracts

**Figure 3:**
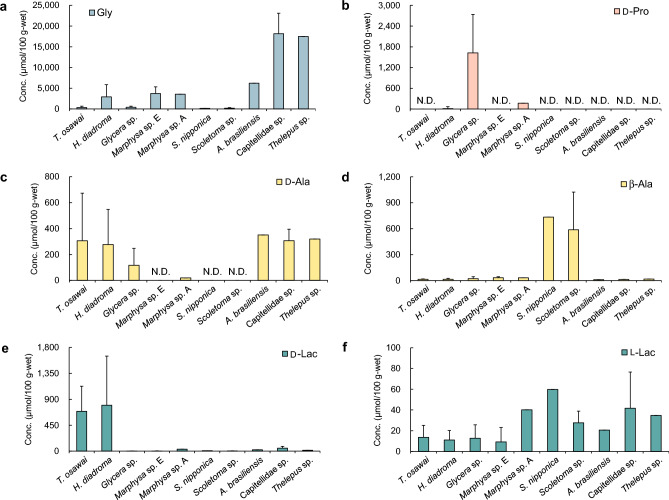
Concentrations of components in the polychaete extracts (mean ± SD [µmol/100 g-wet]). Gly (**a**), d-Pro (**b**), d-Ala (**c**), β-Ala (**d**), d-Lac (**e**), and l-Lac (**f**).

We calculated and compared the concentrations (mean ± SD) of the components present in the extract of each polychaete species without considering the periods and sites of sample collection to identify the characteristics of polychaete extract components. The concentrations of amino acids that act as osmolytes in various marine organisms^25,26^ are shown in Fig. 3, while those of the other amino acids are shown in Supplementary Fig. S4-1–4. The ratio of each amino acid to the total amino acids is shown in Supplementary Fig. S5, and the ratios of d-amino acids to d,l-amino acids are shown in Supplementary Fig. S6.

The concentration of glycine (Gly) was in the range 146–18,154 µmol/100 g-wet (Fig. 3a). Capitellidae sp. and *Thelepus* sp. contained particularly high concentrations of Gly, comprising approximately 80% of their total amino acid contents (Supplementary Fig. S5). d-Pro was detected only in three species of polychaetes: *Glycera* sp. (1625 µmol/100 g-wet, d% = 94), *Marphysa* sp. A (168.2 µmol/100 g-wet*,* d% = 84), and *H. diadroma* (13.88 µmol/100 g-wet*,* d% = 2.3, Fig. 3b and Supplementary Fig. S6). Conversely, l-Pro was detected in the extracts of all polychaetes in the range 31–1421 µmol/100 g-wet (Supplementary Fig. S4-2c). The concentrations of d-Ala and l-Ala in the polychaete extracts ranged from N.D. to 351 (Fig. 3c) and from 462 to 1486 µmol/100 g-wet (Supplementary Fig. S4-1c), respectively. The ratio of d-Ala to the total Ala ranged from 0 to 40% (Supplementary Fig. S6b). High concentration of β-Ala was detected in *S. nipponica* (733 µmol/100 g-wet) and *Scoletoma* sp. (587 µmol/100 g-wet, Fig. 3d). The d-Lac and l-Lac concentrations in the *T. osawai* extract were 691 and 14 (d% = 98), respectively, while those in the *H. diadroma* extract were 797 and 11 µmol/100 g-wet (d% = 99), respectively (Fig. 3e and f and Supplementary Fig. S6f). The d-Lac and l-Lac concentrations in the other polychaetes ranged from 2.00 to 51.3 and from 9.13 to 59.7 µmol/100 g-wet, respectively (Fig. 3e,f), and the ratio of d-Lac to total Lac ranged from 11 to 55% (Supplementary Fig. S6f).

### Cluster and SIMPER analysis

**Figure 4:**
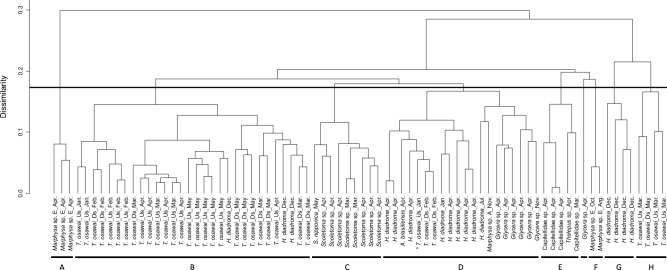
Cluster analysis based on the concentrations of components detected in polychaetes. The organism marked with * was excluded from the hierarchical clustering heatmap analysis. See the *Statistical analysis* section for details. Cluster analysis was performed using the R software (version 4.2.1, https://www.r-project.org/).

We collected 78 polychaetes and divided them into eight groups (Groups A–H) by conducting cluster analysis using the concentrations of the components detected in each organism. The differences among the groups were statistically significant (Fig. 4, *P* = 0.002). Groups B (*T. osawai*), C (*S. nipponica* and *Scoletoma* sp.), and E (Capitellidae sp. and *Thelepus* sp.) were relatively well arranged. The results of SIMPER analysis are shown in Supplementary Table S3. A comparison of Groups B and C, between Groups B and E, and between Groups C and E showed that d-Lac in Group B, d-Ala and β-Ala in Group C, and Gly and d-Asn in Group E exhibited high contribution ratios.

### Seasonal variations in the components of the *T. osawai *extracts

**Figure 5:**
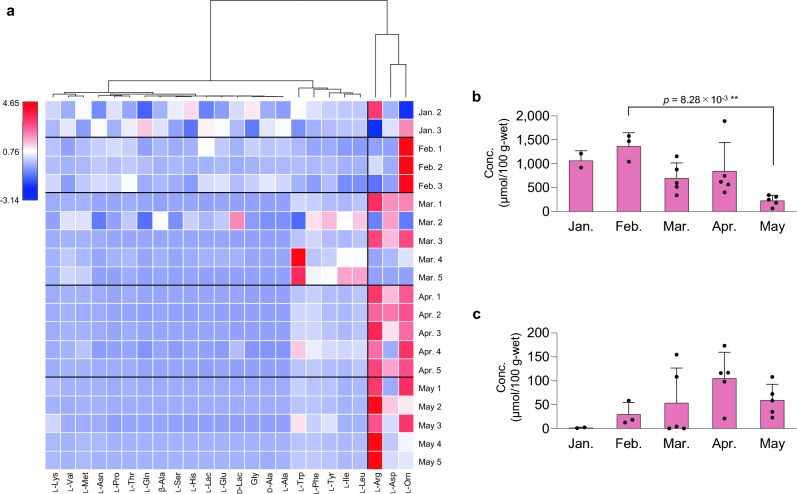
(**a**) Hierarchical clustering heatmap. Horizontal axis: components detected in the extract of *T. osawai*; vertical axis: *T. osawai* collected monthly. Hierarchical clustering heatmap analysis was performed using the R software (version 4.2.1, https://www.r-project.org/). Changes in the concentrations of d-Lac (**b**) and l-Orn (**c**) in the extracts of *T*. *osawai* collected between January and May 2023 (mean ± SD [µmol/100 g-wet]).

Mature *T. osawai* was present in the upstream region of the Arakawa River between late January and late February 2023. The degree of increase in the proportion of each component in the extract of *T. osawai* is shown in Fig. 5a. Between February and May, the proportions of l-Orn, l-Asp, and l-Arg increased more markedly than those of the other components. However, the degree of the increase varied considerably in each sampling month. The concentration of d-Lac increased in February and then decreased gradually (Fig. 5b), whereas that of l-Orn increased from February to May (Fig. 5c). The changes in the concentrations of the other components are shown in Supplementary Fig. S7.

### Effects of different habitats on the components of the *T. osawai* extract

The up- and downstream salinities are 3 and 8 PSU in February and May 2023, respectively, i.e., the salinity in the upstream region is 10% of that of seawater. This area is the upper limit of the tidal area. Even the brackish water clam *Corbicula japonica*, which exhibits a high tolerance toward freshwater, does not inhabit this area. Conversely, the salinity in the downstream region is approximately threefold higher than that in the upstream region. Approximately 21–51 individuals were collected within a 25-cm quadrat upstream, with an average weight of 0.67 ± 0.26 g per individual and an average biomass of 3870 ± 1936 g/m^2^. Furthermore, 20–39 individuals were collected downstream, with an average weight of 0.36 ± 0.15 g per individual and an average biomass of 2446 ± 368 g/m^2^ (Supplementary Table S4).

**Figure 6:**
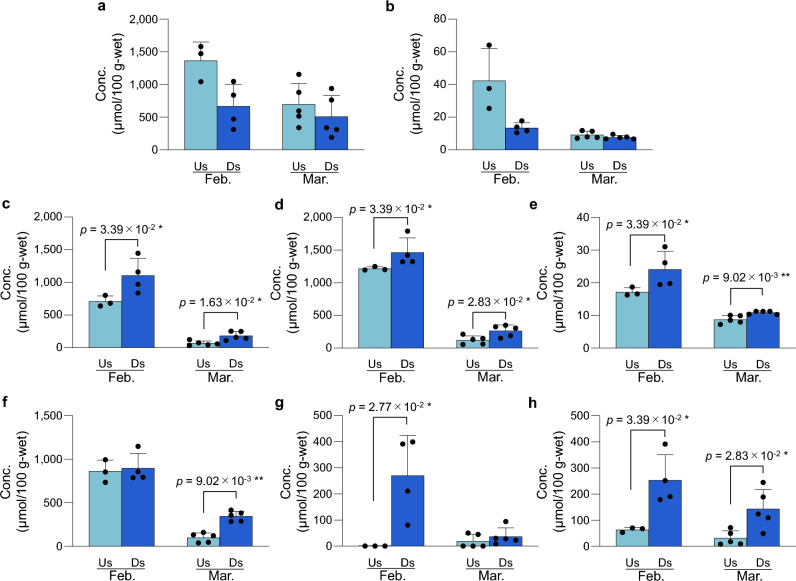
Lac and amino acid concentrations in the extracts of *T*. *osawai* collected from the up- and downstream regions of the Arakawa River (mean ± SD [µmol/100 g-wet]). Horizontal axis: sampling sites, with Us: upstream region and Ds: downstream region, and the month of sample collection in 2023. The concentrations of Lac and amino acid in each organism are indicated by a dot. d-Lac (**a**), l-Lac (**b**), d-Ala (**c**), l-Ala (**d**), β-Ala (**e**), Gly (**f**), l-Asp (**g**), and l-Pro (**h**).

The d- and l-Lac concentrations in the extracts of *T. osawai* collected upstream tended to be higher than those collected downstream (Fig. 6a,b). Conversely, the concentrations of six amino acids (d-Ala, l-Ala, β-Ala, Gly, l-Asp, and l-Pro) were significantly higher in the samples collected downstream (Fig. 6c–h). Amino acids exhibiting no significant differences in concentration between the samples collected up- and downstream are shown in Supplementary Fig. S8.

## Discussion

This study is the first to reveal the concentrations of d- and l-amino acids in polychaetes inhabiting estuaries and inner bays by LC–MS/MS using an amino group-derivatization reagent, CIMa-OSu^19^. In contrast to their concentration in bivalves^1^ and crustaceans^2–4^, there is little information regarding the concentrations and roles of these amino acids in polychaetes. We determined the concentrations of d- and l-Lac in polychaetes owing to the scarcity of available data. Based on these quantitative data on the d- and l-amino acids and d- and l-Lac, we discussed the physiological functions of amino acids and Lac of polychaetes inhabiting areas that exhibit large environmental changes.

Polychaetes contained 27 amino acids, including Gly and five d-amino acids (d-Asn, d-Ala, d-Ser, d-Asp, and d-Pro), which are non-essential amino acids known as osmolytes that can be synthesized by marine organisms (Supplementary Fig. S9)^25,26^. We detected d-Pro only in three species of polychaetes (*H. diadroma*, *Glycera* sp., and Capitellidae sp.). Notably, *Glycera* sp. contained an extremely high concentration of d-Pro, with a higher d% than those exhibited by other marine organisms^1^, such as bivalves (6–238 µmol/100 g-wet), shrimps (0.6–3.7 µmol/100 g-wet), and crabs (3.0–12 µmol/100 g-wet). Although in 2004, Hoeger et al. reported high concentrations of β-Ala in *Nereis japonica*^27^, β-Ala was present in *S. nipponica* and *Scoletoma* sp. The three species of polychaetes, i.e., *H. diadroma*, *Glycera* sp., and Capitellidae sp., collected from the same sites (artificial tidal flat and around Setojima Island within the Mangoku-ura Lagoon) during approximately the same period (March to May 2023) contained low concentrations of β-Ala. Therefore, the high concentration of β-Ala in *S. nipponica* and *Scoletoma* sp. may not be because of the specificity of the collection site. Instead, the abundant β-Ala content may be attributed to the biochemical characteristics of *Scoletoma* sp. *Perinereis aibuhitensis*, a member of the Nereididae family, like *T. osawai* and *H. diadroma*, exhibits considerably high concentrations of d-Ala (3810 µmol/100 g-wet)^26^. Perhaps, *P. aibuhitensis* prefers areas with high salinity (approximately 20 PSU)^28–33^, as compared to *T. osawai* and *H. diadroma* (approximately between 1 and 10 PSU)^34,35^. Taken together with findings of Abe et al*.*^26^, our data suggest that d-Ala concentrations in polychaetes, within the same family, may differ significantly depending on their salinity preferences. Even compared to those of other marine organisms, such as crustaceans and bivalves (d-Ala: 343–2980 µmol/100 g-wet, l-Ala: 554–2358 µmol/100 g-wet, d% = 38–64)^25,26^, the d-Ala concentrations were lower in the polychaete species investigated in this study. Moreover, *T. osawai* and *H. diadroma* contained approximately 13–345-fold higher d-Lac concentrations than those of the other polychaetes. The extremely high d-Lac to d,l-Lac ratio (above 98%, Supplementary Fig. S6f) was not previously reported in a living species. Our observation of the burrows of *T. osawai* revealed holes opening on the surface of the muddy sediment (Fig. 1c,e) and light brown (oxidized) layers lining the burrows (Fig. 1c,d)^36^. Similar oxidized layers were also observed in the burrows of *H. diadroma*. During high tide, these holes supply oxygen through seawater and river water to *T. osawai*. Therefore, the possibility that only anaerobic metabolism is responsible for the high concentrations of d-Lac cannot be ruled out. In summary, a comparative analysis of 10 polychaete species revealed that some polychaete species have extremely high levels of amino acids and show considerable differences in the concentration and composition of d- and l-amino acids and Lac among species, which are large enough to be reflected in the dendrograms.

The next topic is *T. osawai*, which is characterized by extremely high concentrations of d-Lac, and we investigated the monthly variation of the d- and l-amino acids and Lac. *T. osawai* collected between January and May 2023 exhibited monthly variations in its d-Lac concentration, which increased in February and then gradually decreased. Hierarchical clustering heatmap analysis revealed that similar changes were observed in the concentrations of d-Lac, d-Ala, l-Ala, β-Ala, and Gly. *T. osawai* and *H. diadroma* exhibit reproductive swarming^37,38^, i.e., both mature males and females of these two polychaete species swim together at night at high tide, release eggs or sperms into the water, and subsequently die^37^. From an ecological perspective, *T. osawai*, which generally lives in burrows, requires considerable energy to get out of the burrow to swim and mate with other individuals. d-Lac and several amino acids may be involved in acquiring and storing energy for their living.

We found a seasonal alteration in l-Orn concentration in *T. osawai*. The proportion of l-Orn in *T. osawai* began to increase gradually in February, followed by a notable increase in April. l-Orn is an amino acid associated with the urea cycle^39^. However, limited data are available on nitrogen metabolism in marine invertebrates, including polychaetes such as *T. osawai*. Further studies on urea cycle-related components, such as ammonia and urea, should be conducted to obtain further insights into the nitrogen metabolism of *T. osawai* to determine the presence and seasonal variation of l-Orn in *T. osawai*.

Next, we focused on the salinity of the habitat of *T. osawai*. Both the weight of one individual and the amount of biomass per unit area tended to be higher upstream than downstream (Supplementary Table S4). The upstream region away from the mouth of the river, where *T. osawai* is abundant, has low salinity and is thus unsuitable for many marine organisms. Similar results have been reported by Hanafiah et al.^28^. The d-Lac concentration of *T. osawai* found in the upstream region is higher than that occurring in the downstream region, strongly suggesting that d-Lac might have a meaningful role in upstream habitation. Consequently, *T. osawai* may exhibit a different “osmotic adaptation system,” compared to that demonstrated by other organisms. While amino acids in other marine organisms help adapt to high salinity, d-Lac in *T. osawai* may be involved in its adaptation to low-salinity conditions. Therefore, we posit that d-Lac in *T. osawai* is utilized as an energy source for reproduction and is involved in adaptation to low-salinity upstream regions. These characteristics of *T. osawai* may be responsible for the expansion of its distribution area to specific niches.

We conclusively obtained highly reliable data regarding d-amino acids in polychaetes based on an LC–MS/MS analysis with unique fragmentation^19^. We found that a polychaete, *T. osawai* contained notable amounts of d-Lac. Component analysis of amino acids and Lac enantiomers can provide novel perspective regarding the life histories of polychaetes and their responses to environmental variations.

In future studies, we intend to perform exhaustive sampling in different seasons and environments to clarify the characteristics of the distributions of the d-forms and their functions in polychaetes. In addition to increasing the number of polychaete species, investigating changes in the concentrations and compositions of amino acids and Lac, along with the life histories from juvenile to mature, should be highly informative, particularly in elucidating the physiological functions of amino acids and Lac enantiomers in polychaetes.

### Supplementary Information

Supplementary Information.

## Data Availability

All data analyzed during this study are included in the published article and its Supplementary Information files.
