# Supplementary material for: Characterization of polychaetes inhabiting estuaries and inner bays by composition analysis of amino acids and lactate enantiomers
Source: Sci Rep. 2024 Mar 6;14:5494. doi: 10.1038/s41598-024-55861-5 (PMC10918092; doi:10.1038/s41598-024-55861-5)
Supplement: Supplementary file 1 — Supplementary Information. [file 41598_2024_55861_MOESM1_ESM.docx]

*Supplementary Information*

**Characterization of polychaetes inhabiting estuaries and inner bays by composition analysis of amino acids and lactate enantiomers**

Mayu Onozato^1^, Wataru Shinohara^2^, Yuichiro Osaka^3^, Tatsuya Sakamoto^1^, Maho Umino^1^, Atsuko Nishigaki^3^, Kenji Okoshi^3^, Takeshi Fukushima^1^

^1^ *Department of Analytical Chemistry, Faculty of Pharmaceutical Sciences, Toho University, 2-2-1 Miyama, Funabashi-shi, Chiba 274-8510, Japan*

^2^ *Chiba Municipal Chiba High School, 9-46-1 Konakadai, Inage-ku, Chiba-shi, Chiba 263-0043, Japan*

^3^ *Department of Environmental Science, Faculty of Science, Toho University, 2-2-1 Miyama, Funabashi-shi, Chiba 274-8510, Japan*

**Correspondence.** Mayu Onozato, Ph.D.

Department of Analytical Chemistry, Faculty of Pharmaceutical Sciences, Toho University, 2-2-1 Miyama, Funabashi-shi, Chiba 274-8510, Japan

*Tel./Fax:* 81-47-472-1523; *E-mail:* mayu.onozato@phar.toho-u.ac.jp

**Contents**

|  |  | |  | |  |
| --- | --- | --- | --- | --- | --- |
| **1.** | Chemicals and reagents | | | | Page 3 |
| **2.** | Pretreatment of homogenates for amino acid analysis | | | | Page 4 |
| **3.** | LC-MS/MS analysis for amino acids derivatized with (*R*)-CIMa-OSu | | | | Page 4 |
| **4.** | Pretreatment of homogenates for Lac analysis | | | | Page 5 |
| **5.** | Column-switching HPLC-fluorescence detection method for derivatized Lac with NBD-PZ | | | | Page 6 |
|  |  | |  | |  |
| **6.** | Supporting Figures | | | |  |
|  | 6. 1 | Fig. S1 | Sampling sites. | | Page 8 |
|  | 6. 2 | Fig. S2 | Chromatogram of Ala within the extract of *Scoletoma* sp. obtained using the mixed-phase column after derivatization with CIMa-OSu (a). Negative CID mass spectrum of the CIMa derivatives of the primary amino acid β-Ala (b). | | Page 9 |
|  | 6. 3 | Fig. S3 | Chromatograms of D,L-Lac obtained using an ODS column (a), and enantiomeric separation of D-Lac and L-Lac using a chiral column (b). (i) 500 μM D,L-Lac and (ii) 250 µM D-Lac. | | Page 10 |
|  | 6. 4 | Fig. S4 | Concentrations of amino acids in the polychaete extracts (mean ± SD [µmol/100 g-wet]). | | Page 11 |
|  | 6. 5 | Fig. S5 | Amino acid compositions (%) of the polychaete extracts. | | Page 15 |
|  | 6. 6 | Fig. S6 | D-Amino acid contents (%) of the polychaete extracts. | | Page 16 |
|  | 6. 7 | Fig. S7 | Changes in the concentrations of amino acids in the extracts of *T*. *osawai* collected between January and May 2023 (mean ± SD [µmol/100 g-wet]). | | Page 17 |
|  | 6. 8 | Fig. S8 | Amino acid concentrations in the extracts of *T*. *osawai* collected from the up- and downstream regions of the Arakawa River (mean ± SD [µmol/100 g-wet]). | | Page 20 |
|  | 6. 9 | Fig. S9 | Biosynthetic pathways of amino acids utilized as osmolytes in aquatic invertebrates. | | Page 22 |
|  |  | |  | |  |
| **7.** | Supporting Tables | | | |  |
|  | 7. 1 | Table S1 | Number of polychaetes collected from each site. | | Page 23 |
|  | 7. 2 | Table S2 | Biological characteristics of polychaetes investigated in this study. | | Page 24 |
|  | 7. 3 | Table S3 | Results of similarity of percentages (SIMPER) analysis. | | Page 25 |
|  | 7. 4 | Table S4 | Biomass estimation of *T. osawai* in the Arakawa River. | | Page 26 |
|  | | | |  | |
| **References** | | | |  | |

**1. Chemicals and reagents**

l-Alanine (Ala), l-arginine (Arg), l-asparagine (Asn), l-aspartic acid (Asp), l-citrulline (Cit), l-glutamine (Gln), l-glutamate (Glu), glycine (Gly), l-histidine (His), l-isoleucine (Ile), l-leucine (Leu), l-lysine (Lys), l-methionine (Met), l-ornithine (Orn), l-phenylalanine (Phe), l-proline (Pro), l-serine (Ser), l-tryptophan (Trp), l-threonine (Thr), l-tyrosine (Tyr), and l-valine (Val) were obtained from Kyowa Hakko Bio (Tokyo, Japan). d-Ala, d-Phe, d-Trp, d-Ser, high-performance liquid chromatography (HPLC)-grade CH_3_OH (MeOH), liquid chromatography-mass spectrometry (LC-MS)-grade MeOH, HPLC-grade formic acid, and APDSTAG^®^ Wako Amino Acids Internal Standard Mixture Solution were obtained from FUJIFILM Wako Pure Chemical (Osaka, Japan). 4-Dimethylaminopyridine (DMAP), triphenylphosphine (TPP), 2,2′-dipyridyl disulfide (DPDS),β-Ala, d-Arg, d-Asn, D-Asp, d-Gln, d-Glu, d-His, d-Ile, d-Leu, d-Lys, d-Thr, and d-Val were purchased from Tokyo Chemical Industry (Tokyo, Japan). d-Kynurenine (KYN), l-KYN, d-Met, d-Tyr, dl-Orn, d-Pro, sodium d-lactate (d-Lac), sodium l-lactate (l-Lac), and ammonium formate were procured from Sigma-Aldrich (St. Louis, MO, USA). dl-Cit was purchased from Matrix Scientific (Columbia, SC, USA), HPLC-grade CH_3_CN and LC-MS-grade CH_3_CN were obtained from Kanto Kagaku (Tokyo, Japan), and phosphate-buffered saline (PBS) was purchased from Nissui (Tokyo, Japan). The water used was purified using a Milli-Q Lab system (Nihon Millipore, Tokyo, Japan). Millex^®^-LG filters (0.20 μm) were purchased from Merck (Darmstadt, Germany).

**2. Pretreatment of homogenates for amino acid analysis**

The thawed sample (five-fold diluted homogenate of polychaete (10 µL) and CH_3_CN/MeOH (1:1, *v*/*v*, 130 µL)) was mixed with an internal standard mixture (10 µL), vortexed for 1 min, and centrifuged at 2,500 × *g* for 5 min at 4 ℃. The supernatant (130 μL) was evaporated under reduced pressure using a centrifugal evaporator. H_2_O (10 μL) was added to the residue and vortexed for 1 min. Subsequently, 20 mM of (*R*)-succinimidyl 2-(3-[(benzyloxy)carbonyl]-1-methyl-5-oxoimidazolidin-4-yl)acetate ((*R*)-CIMa-OSu) in CH_3_CN (10 μL) and 30 mM of DMAP in CH_3_CN (10 μL) were added. The solution was vortexed vigorously for 1 min and allowed to react for 60 min at room temperature (approximately 22 ℃). Subsequently, 0.1 % formic acid in CH_3_CN (1.0 mL) was added to the solution to stop the reaction. The resultant solution was subjected to solid-phase extraction (SPE) using an InertSep^®^ NH_2_ SPE cartridge (GL Sciences, Tokyo, Japan), as described in our previous study for the miso sample [1]. The final eluate (100 μL) was mixed with the mobile phase A/B (9/1, *v*/*v*, 100 μL; A and B are defined in Section 3 below) and filtered using Millex^®^-LG filters (0.20 μm). The filtrate was analyzed by LC-tandem mass spectrometry (LC-MS/MS).

**3. LC**-**MS/MS analysis for amino acids derivatized with (*R*)-CIMa-OSu**

A triple quadrupole LCMS-8040 mass spectrometer (Shimadzu, Kyoto, Japan) attached to an electrospray ionization interface was used in LC-MS/MS. Two pumps (LC-20AD), an autosampler (SIL-20AC) and a column oven (CTO-20A), and PC software (LabSolutions ver. 5.80, Shimadzu) were used. The temperature of the autosampler tray was set at 4 ℃. The analytical column was a Scherzo SS-C18^®^ column (250 × 2.0 mm; i.d., 3 μm, Imtakt, Kyoto, Japan) maintained at 60 ℃ in the column oven. The mobile phase, which was H_2_O/MeOH/10 mM ammonium formate in H_2_O (pH 2.8, 5/2/3, *v*/*v*/*v*) (A) and 10 mM ammonium formate in [H_2_O/MeOH (3/7, *v*/*v*)] (B), was pumped constantly at a flow rate of 0.2 mL/min using the following elution program: (0–20 min) B% = 10; (20.01–56 min) B% = 10–59; (56.01–60 min) B% = 59–100; (60.01–75 min) B% = 100; (75.01–90 min) B% = 10. The injection volume was 3.0 μL. The desolvation line and heatblock temperatures were adjusted to 250 and 400 ℃, respectively. The flow rates of the nebulizing and drying gases, ion-spray voltage, and collision-induced dissociation (CID) gas pressure were the same as those used in our previous study [2], i.e., 3.0 and 10 L/min, 4.5 kV, and 230 kPa, respectively. Ions were detected in the multiple reaction monitoring mode ([M + H]^+^ > 91.1) and were quantified using MS/MS detection in the positive ion mode [2].

**4. Pretreatment of homogenates for Lac analysis**

The thawed sample (five-fold diluted homogenate of polychaete (10 µL) and CH_3_CN/MeOH (1:1, *v*/*v*, 130 µL)) was mixed with H_2_O (10 µL), vortexed for 1 min, and centrifuged at 2,500 × *g* for 5 min at 4 ℃. The supernatant (130 μL) was evaporated under reduced pressure using a centrifugal evaporator. H_2_O (10 μL) was added to the residue and vortexed for 1 min, mixed with 30 mM 4-nitro-7-piperazino-2,1,3-benzoxadiazole (NBD-PZ) in CH_3_CN (10 μL), 250 mM TPP in CH_3_CN (10 μL), and 250 mM DPDS in CH_3_CN (10 μL), and left for 120 min at room temperature. Then, 0.1% formic acid in CH_3_CN/MeOH/H_2_O (12/20/68, *v*/*v*/*v*, 160 μL) was then added to the solution, which was vortexed for 1 min to stop the reaction. Subsequently, the fluorescence-derivatized solution was subjected to SPE using an InertSep SCX (100 mg/mL, GL Sciences) conditioned with CH_3_CN (500 μL), followed by H_2_O (500 μL). Each time a solvent was added, the cartridge was centrifuged at 650 × *g* for 1 min. The sample (200 μL) was then loaded onto the cartridge and centrifuged for 15 min at 1,500 × *g*. The analytes were eluted from the cartridge using 0.1 % formic acid in CH_3_CN/MeOH/H_2_O (12/20/68, *v*/*v*/*v*, 200 μL) by centrifugation at 1,500 × *g* for 15 min. Finally, the eluate was filtered using Millex^®^-LG filters (0.20 μm) and analyzed by HPLC-fluorescence detection [3].

**5. Column-switching HPLC-fluorescence detection method for derivatized Lac with NBD-PZ**

Each sample (10 μL) was injected into the column-switching HPLC system with fluorescence detection. The HPLC system used in this study comprised an autosampler (L-2200, Hitachi, Tokyo, Japan), two pumps (LC-20AD, Shimadzu), a column oven (CTO-10A VP, Shimadzu), and two fluorescence detectors (RF-20A for the octadecyl silica (ODS) column and RF-20Axs for the chiral column, Shimadzu). The chromatograms were obtained and analyzed using CDS Plus ver. 5.0 (LAsoft, Chiba, Japan). A column oven was used to maintain the temperature of the separation column (250 × 4.6 mm; i.d., 5.0 μm; TSKgel^®^ ODS-80Ts QA, Tosoh, Tokyo, Japan) at 35 °C. Mobile phases A and B were 0.05 % formic acid in CH_3_CN/MeOH/H_2_O (12/20/68, *v*/*v*/*v*) and CH_3_CN, respectively. The program for the mobile phase was 0 % B (0–60.0 min) for the isocratic elution of dl-Lac, 100 % B (60.1–75.0 min) to wash the column, and 0 % B (75.1–90.0 min) for initialization at a flow rate of 0.7 mL/min. The detector wavelength was set at 547 nm, with an excitation wavelength of 491 nm. The six-port valve (HV-992-01, Jasco, Tokyo, Japan) was switched manually, and a portion of the fraction containing d- and l-Lac was introduced into the chiral column (150 × 4.6 mm; i.d., 5.0 μm; CHIRALPAK AD-RH, Daicel, Osaka, Japan) via a 100-µL sample loop. The mobile phase for the chiral column was CH_3_CN/H_2_O (60/40, *v*/*v*) at a flow rate of 0.3 mL/min. The detector wavelength was the same as that described above [3].

**6. Supporting Figures**

**6.1**

**FIGURE S1** Sampling sites.

Site # 1: artificial tidal flats within the Mangoku-ura Lagoon; # 2: around of Setojima Island within the Mangoku-ura Lagoon; # 3: estuary of the Tamagawa River; # 4: upstream region of the Arakawa River; # 5: downstream region of the Arakawa River; # 6: Sanbanse tidal flat; # 7: shore of Makuhari; and # 8: Yoro tidal flat.


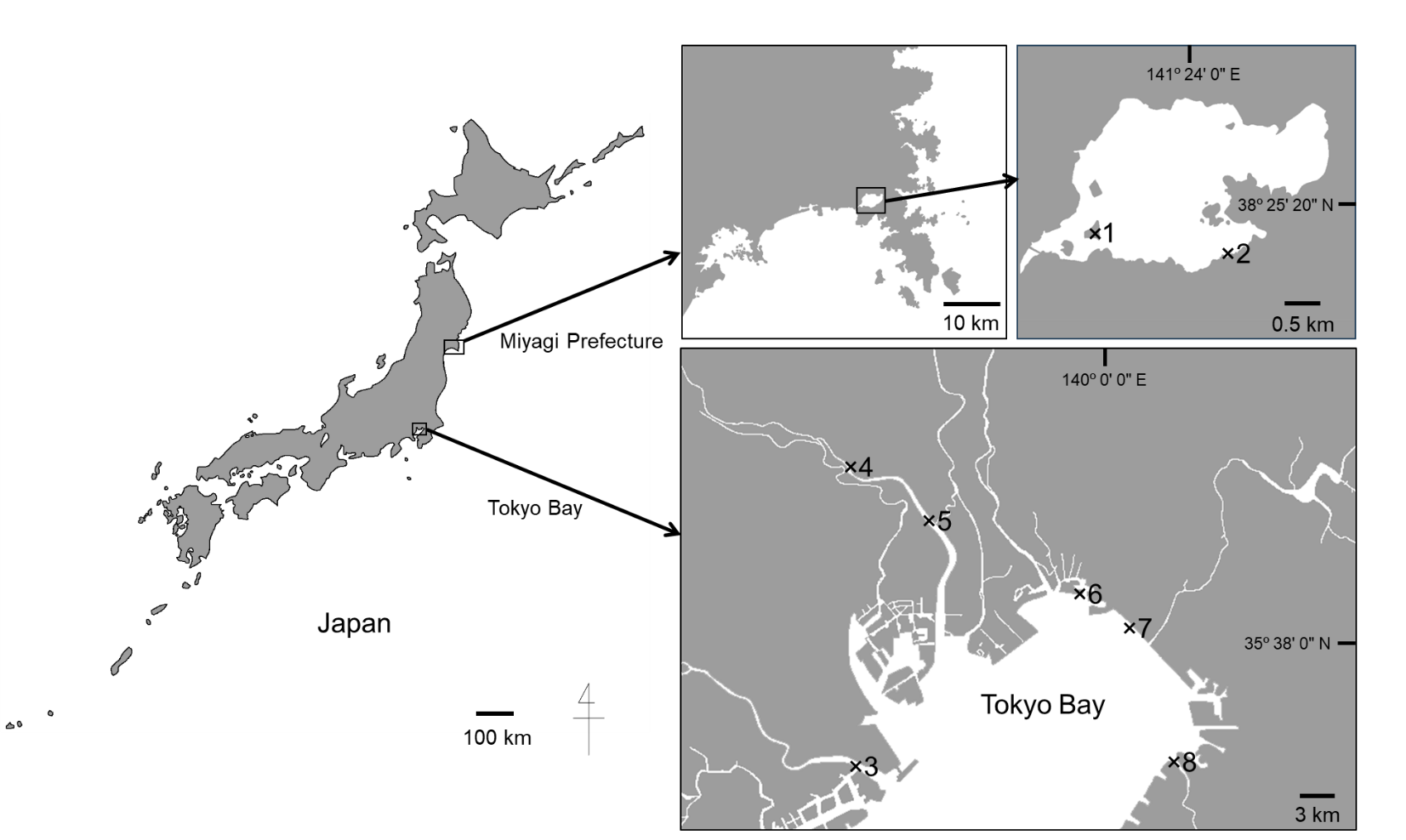


**6.2**

**FIGURE S2** Chromatogram of Ala within the extract of *Scoletoma* sp. obtained using the mixed-phase column after derivatization with CIMa-OSu (a). Negative CID mass spectrum of the CIMa derivatives of the primary amino acid β-Ala (b).

**a**


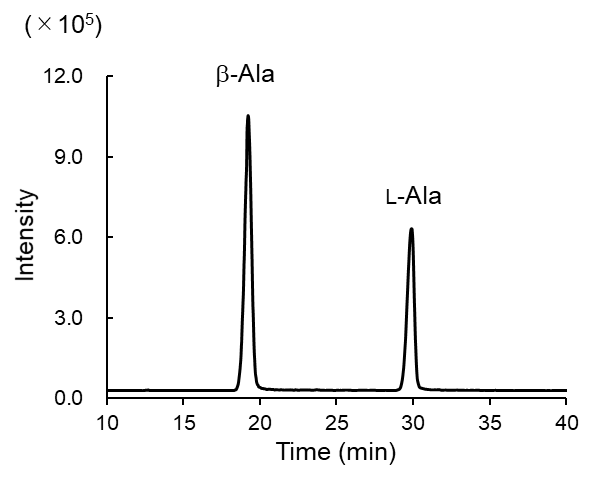


**b**


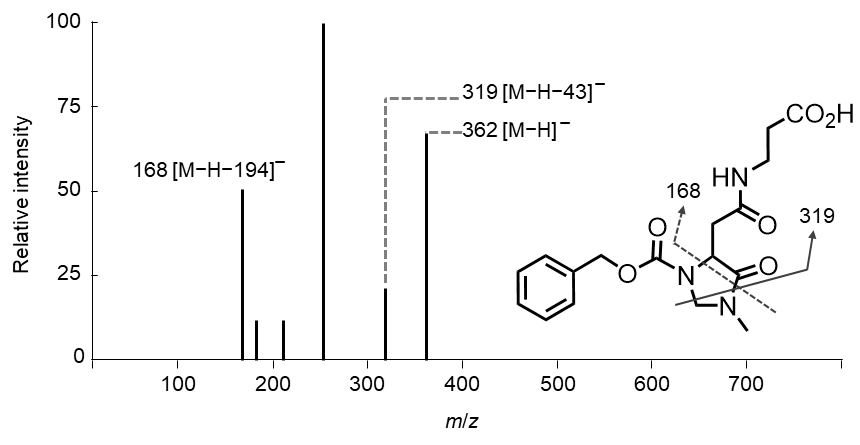


**6.3**

**FIGURE S3** Chromatograms of D,L-Lac obtained using an ODS column (a), and enantiomeric separation of D-Lac and L-Lac using a chiral column (b). (i) 500 µM D,L-Lac and (ii) 250 µM D-Lac.


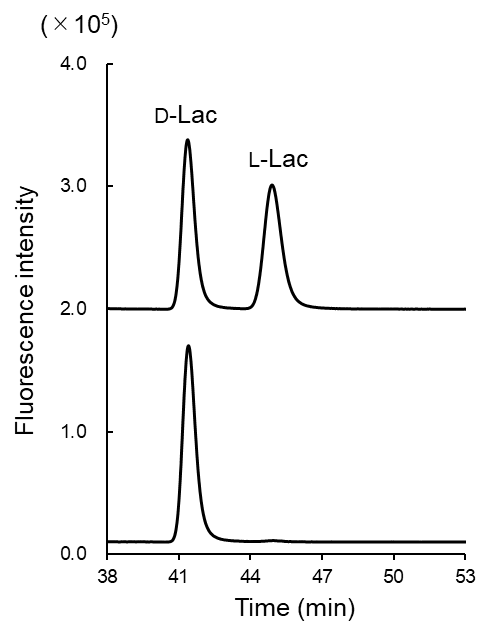


**a**

**b**

(i)

(ii)

(i)

(ii)


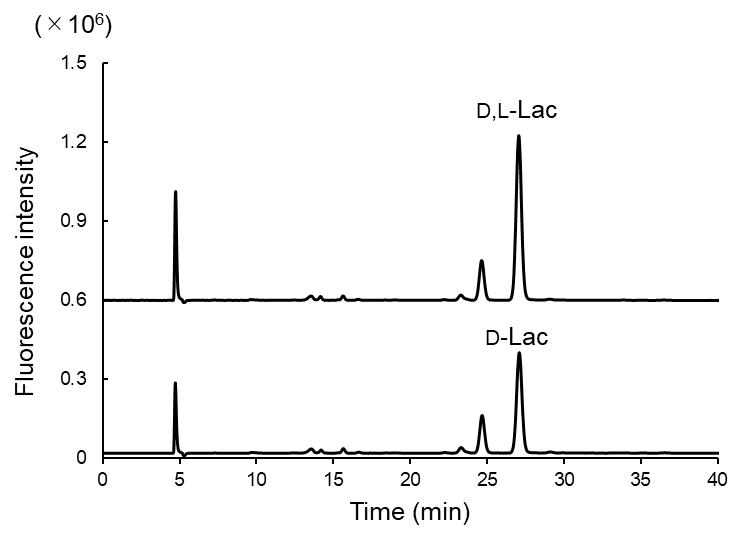


**6.4**

*H. diadroma*

*Marphysa* sp. E

*Glycera* sp.

*S. nipponica*

*Scoletoma* sp.

Capitellidae sp.

*Thelepus* sp.

*T. osawai*

*Marphysa* sp. A

*A. brasiliensis*

**FIGURE S4-1** Concentrations of amino acids in polychaete extracts (mean ± SD [µmol/100 g-wet]). D-Asn (a), L-Asn (b), L-Ala (c), L-Gln (d), D-Ser (e), L-Ser (f), and L-Glu (g).


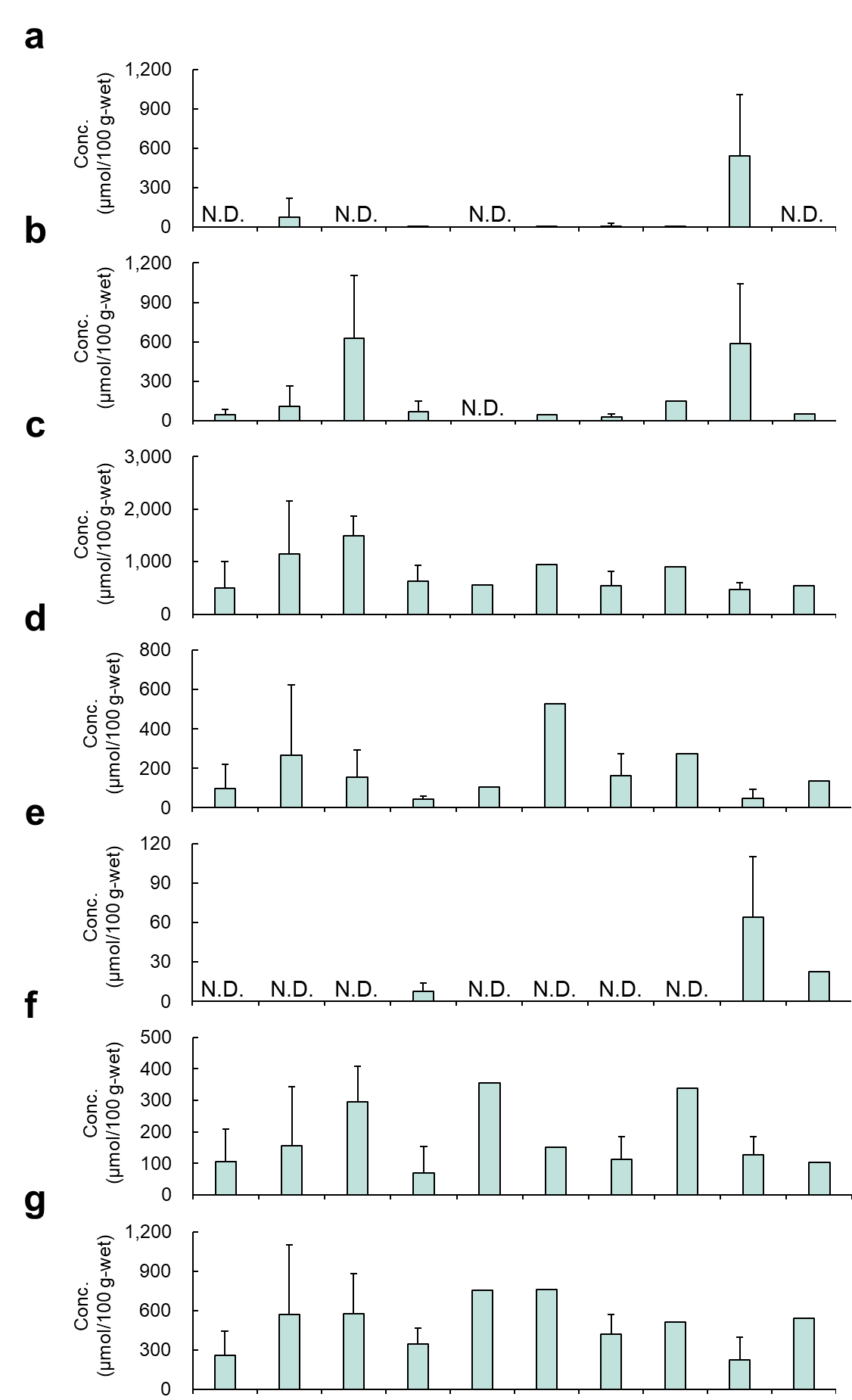


**FIGURE S4-2** Concentrations of amino acids in the polychaete extracts (mean ± SD [µmol/100 g-wet]). D-Asp (a), L-Asp (b), L-Pro (c), L-Arg (d), L-Tyr (e), L-Cit (f), and L-Thr (g).

*H. diadroma*

*Marphysa* sp. E

*Glycera* sp.

*S. nipponica*

*Scoletoma* sp.

Capitellidae sp.

*Thelepus* sp.

*T. osawai*

*Marphysa* sp. A

*A. brasiliensis*


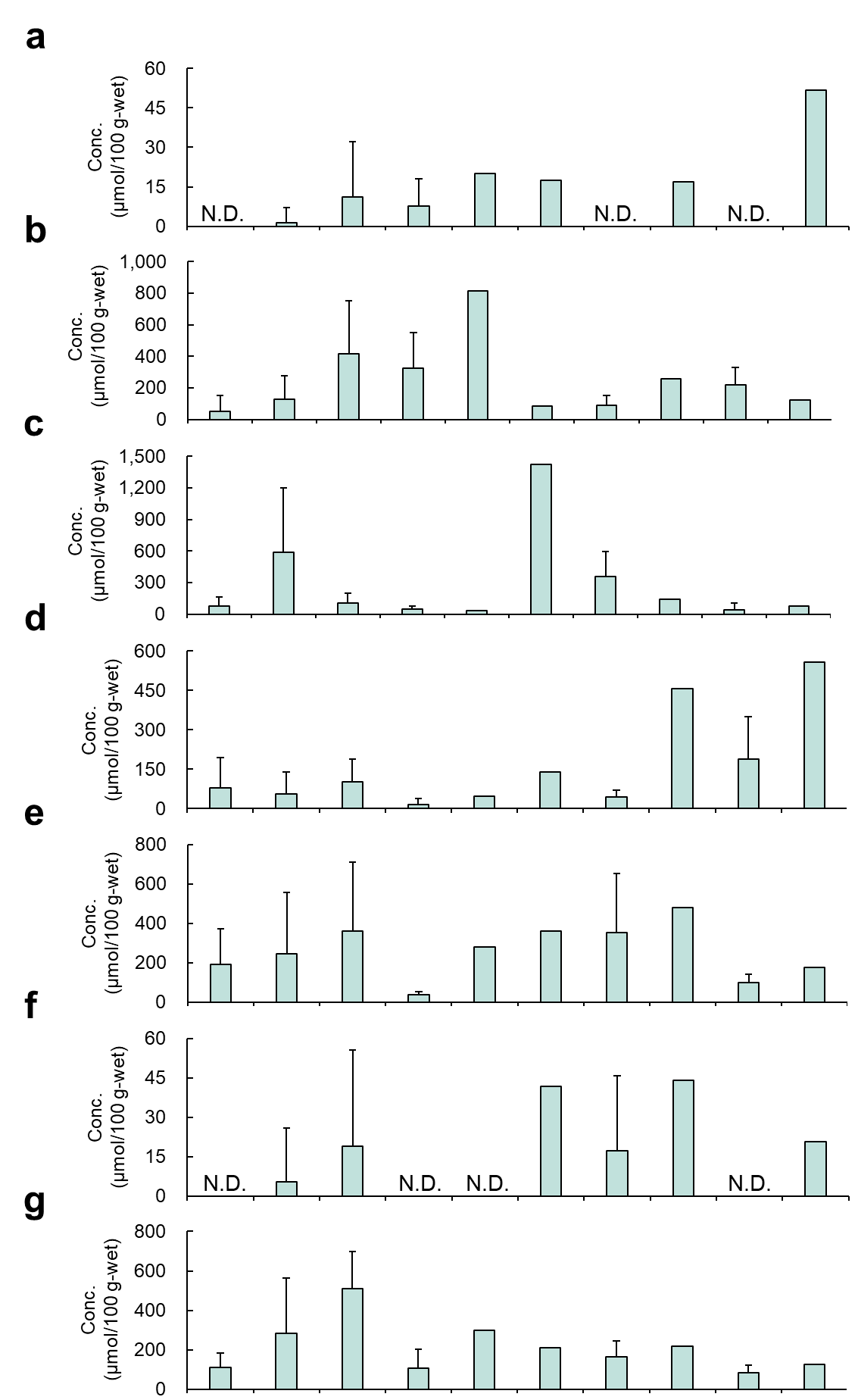


**FIGURE S4-3** Concentrations of amino acids in the polychaete extracts (mean ± SD [µmol/100 g-wet]). L-His (a), L-Val (b), L-Met (c), L-Ile (d), L-Leu (e), L-Trp (f), and L-Phe (g).

*H. diadroma*

*Marphysa* sp. E

*Glycera* sp.

*S. nipponica*

*Scoletoma* sp.

Capitellidae sp.

*Thelepus* sp.

*T. osawai*

*Marphysa* sp. A

*A. brasiliensis*


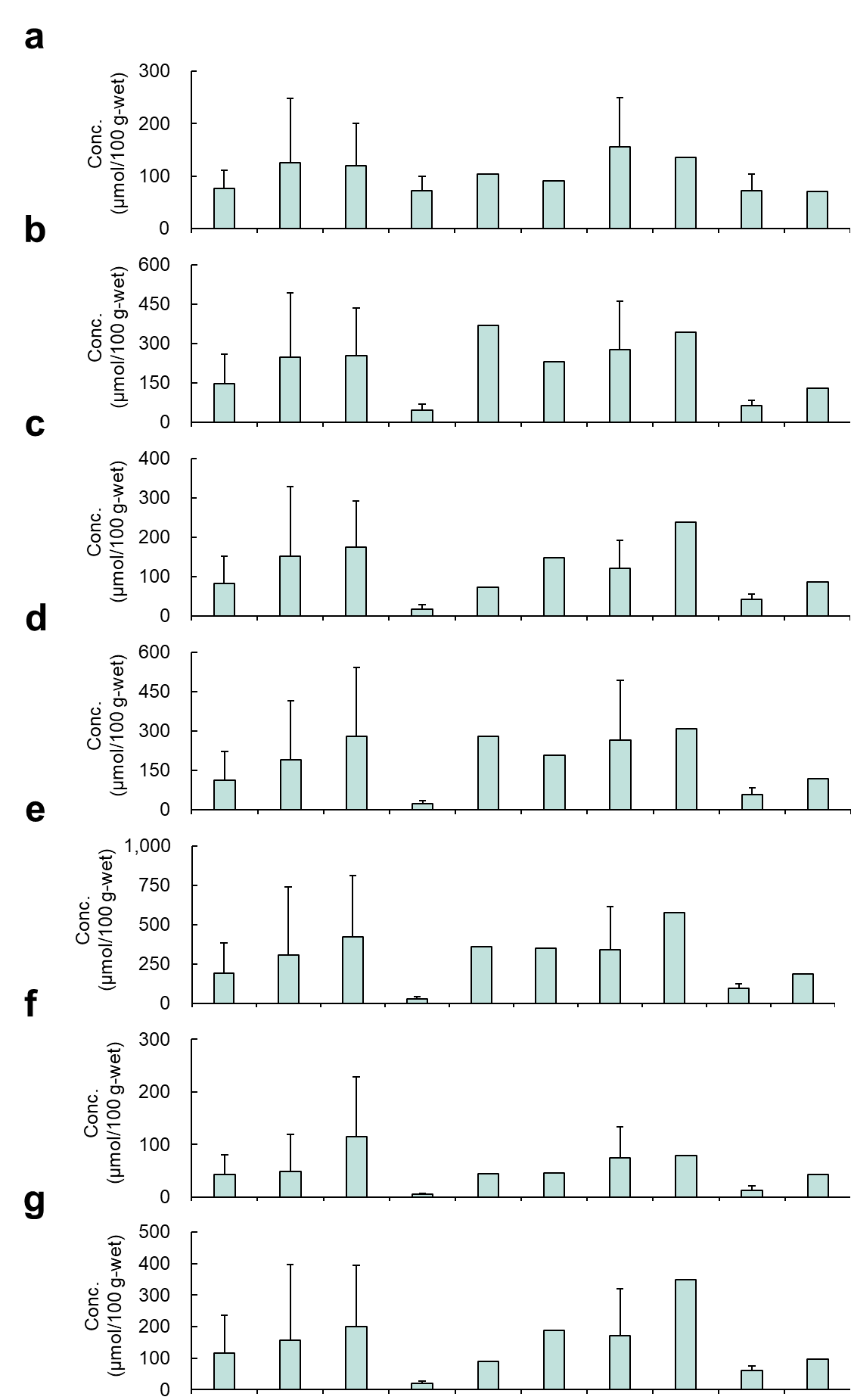


**FIGURE S4-4** Concentrations of amino acids in the polychaete extracts (mean ± SD [µmol/100 g-wet]). L-Lys (a) and L-Orn (b).

*H. diadroma*

*Marphysa* sp. E

*Glycera* sp.

*S. nipponica*

*Scoletoma* sp.

Capitellidae sp.

*Thelepus* sp.

*T. osawai*

*Marphysa* sp. A

*A. brasiliensis*


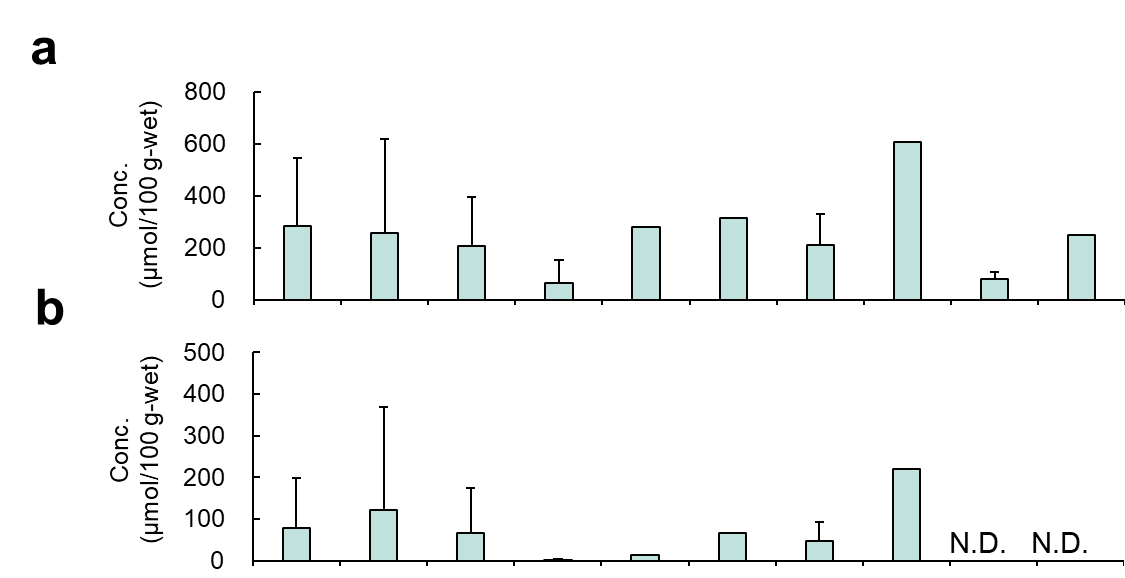


**6.5**

**FIGURE S5** Amino acid compositions (%) of the polychaete extracts.


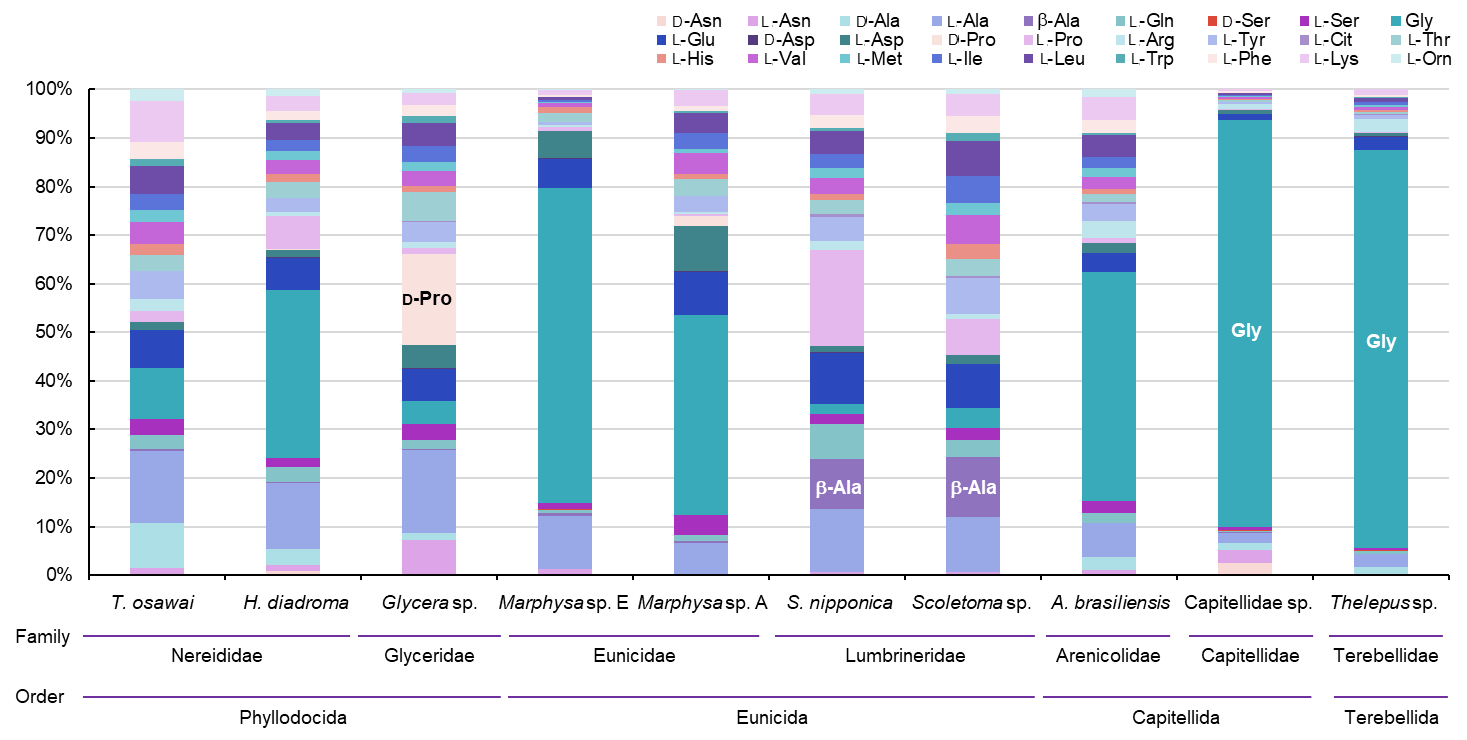


**6.6**

**FIGURE S6** D-Amino acid contents (%) of polychaete extracts.

D-Asn (a), D-Ala (b), D-Ser (c), D-Asp (d), D-Pro (e), and D-Lac (f).

The percentage of the D-amino acid is calculated as the ratio of the D-amino acid concentration to the total (D- and L-) amino acid concentration. The percentage of D-Lac is calculated in the same manner.

*H. diadroma*

*Marphysa* sp. E

*Glycera* sp.

*S. nipponica*

*Scoletoma* sp.

Capitellidae sp.

*Thelepus* sp.

*T. osawai*

*Marphysa* sp. A

*A. brasiliensis*


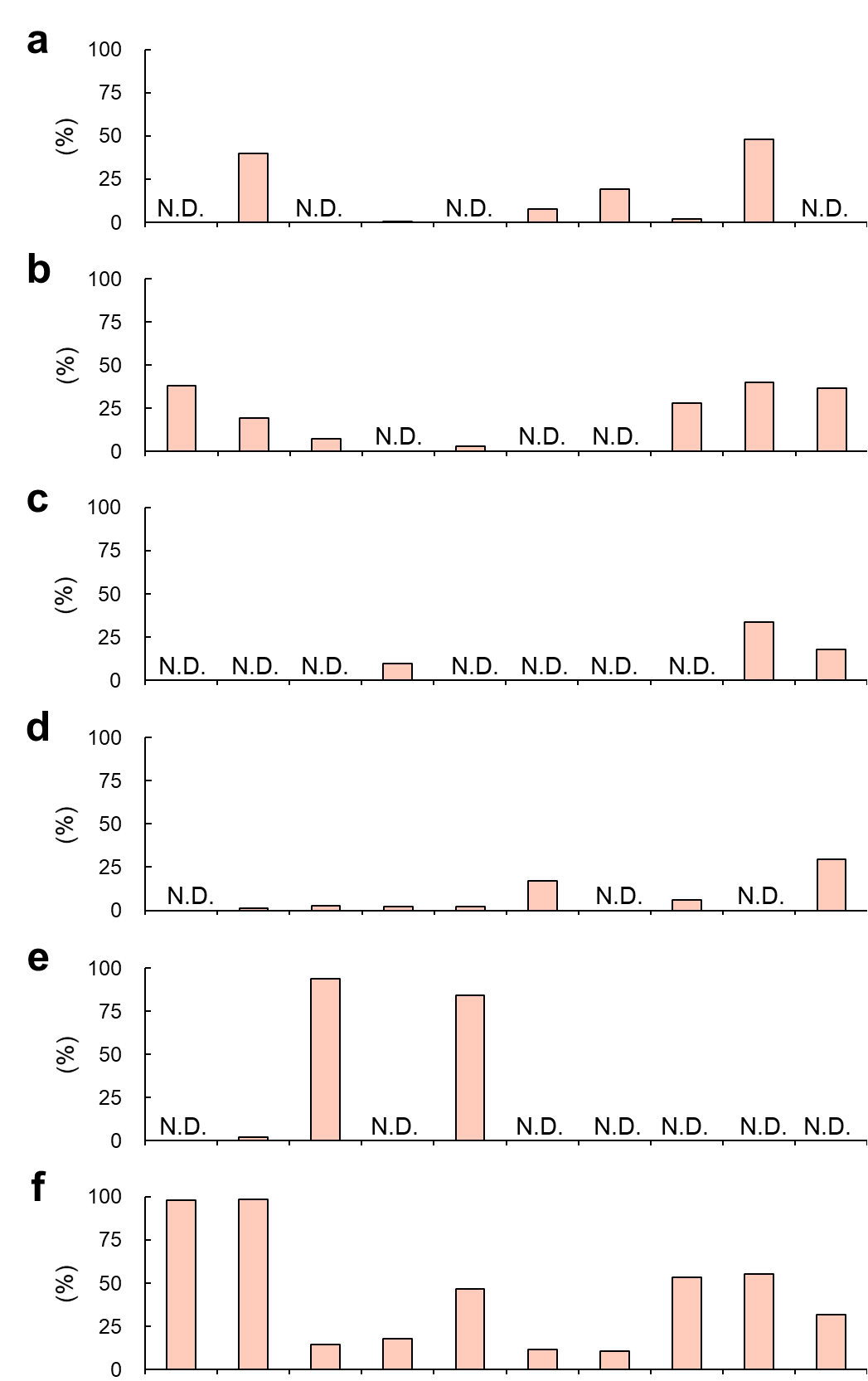


**6.7**

**FIGURE S7-1** Changes in the concentrations of amino acids in the extracts of *T*. *osawai* collected between January and May 2023 (mean ± SD [µmol/100 g-wet]). L-Asp (a), L-Arg (b), L-Leu (c), L-Ile (d), L-Tyr (e), L-Phe (f), L-Trp (g), L-Ala (h), D-Ala (i), and Gly (j).


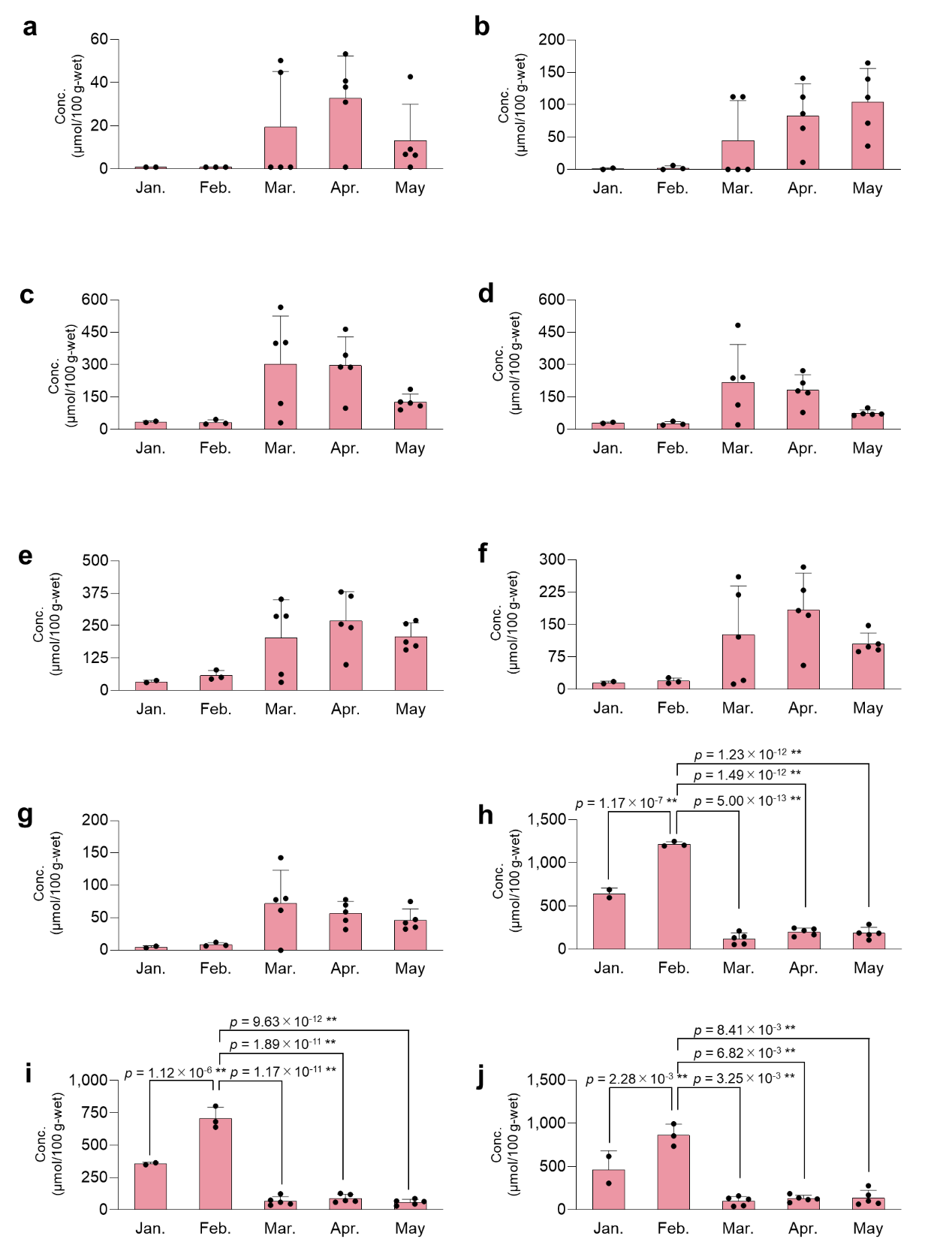


**FIGURE S7-2** Changes in the concentrations of amino acids in the extracts of *T*. *osawai* collected between January and May 2023 (mean ± SD [µmol/100 g-wet]). L-Glu (a), L-Lac (b), L-His (c), L-Ser (d), β-Ala (e), L-Gln (f), L-Thr (g), L-Pro (h), L-Asn (i), and L-Met (j).


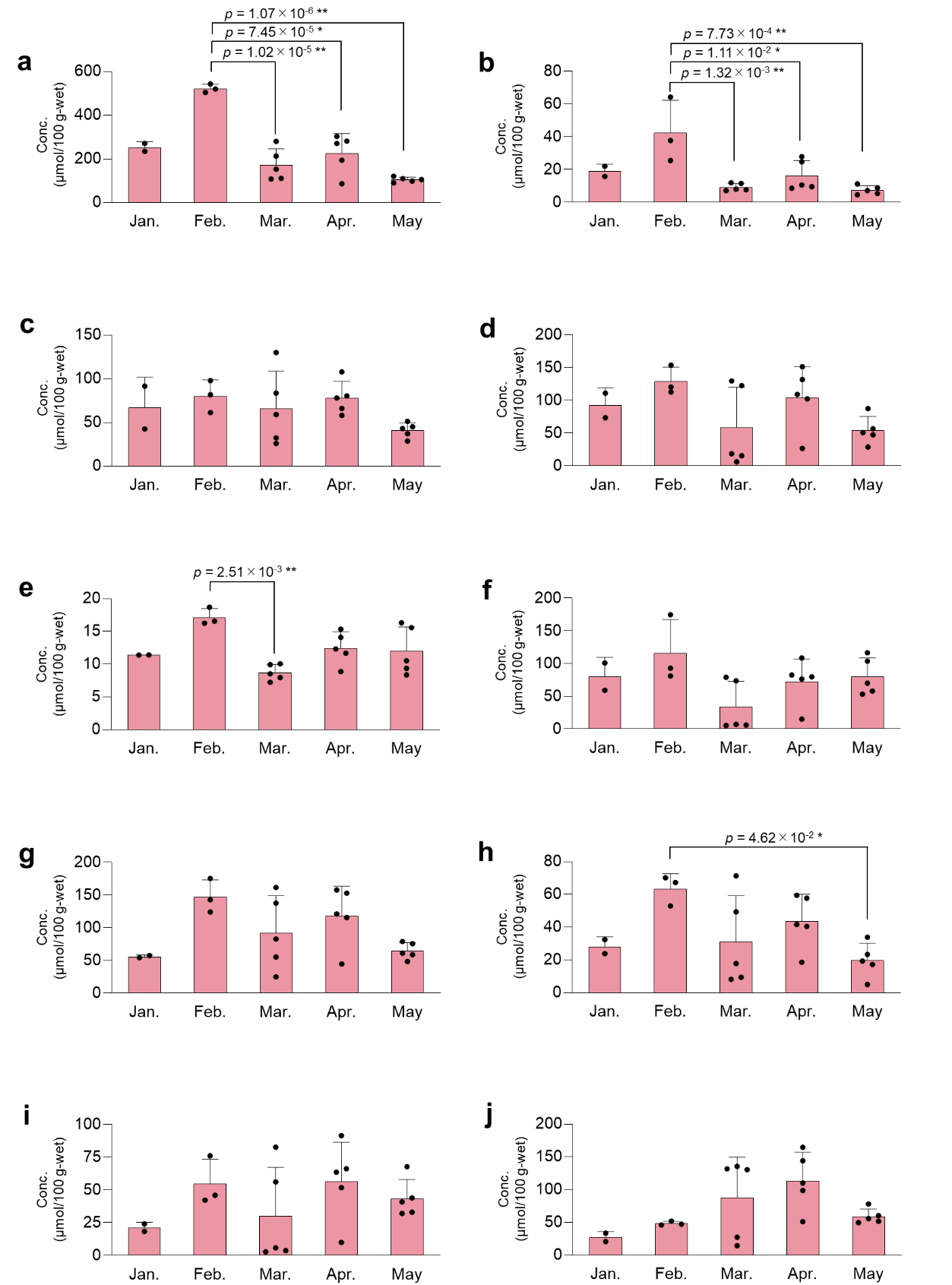


**FIGURE S7-3** Changes in the concentrations of amino acids in the extracts of *T*. *osawai* collected between January and May 2023 (mean ± SD [µmol/100 g-wet]). L-Val (a) and L-Lys (b).


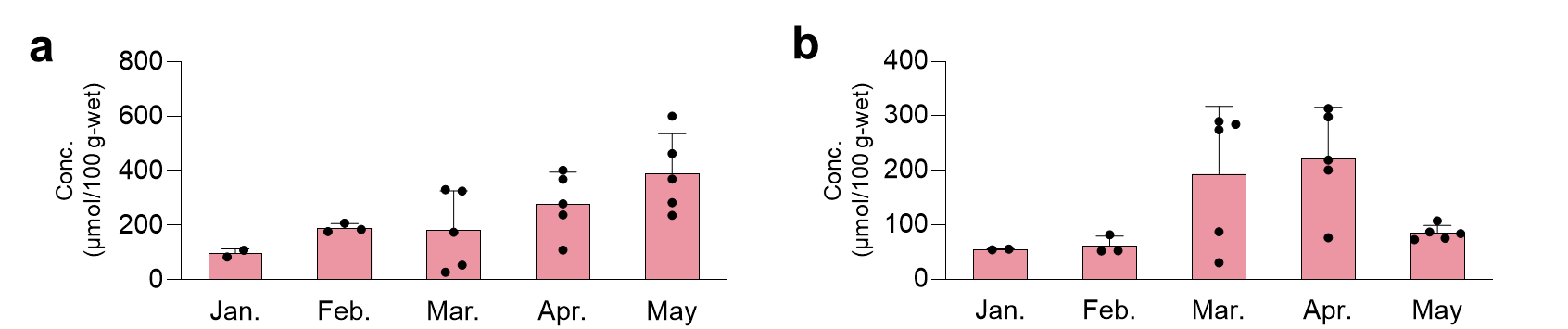


**6.8**

**FIGURE S8-1** Amino acid concentrations in the extracts of *T*. *osawai* collected from the up- and downstream regions of the Arakawa River (mean ± SD [µmol/100 g-wet]). Horizontal axis: sampling sites, with Us: upstream and Ds: downstream, and the month of sample collection in 2023. The concentration in each organism is indicated by a dot. L-Asn (a), L-Gln (b), L-Ser (c), L-Glu (d), L-Arg (e), L-Tyr (f), L-Thr (g), and L-His (h).


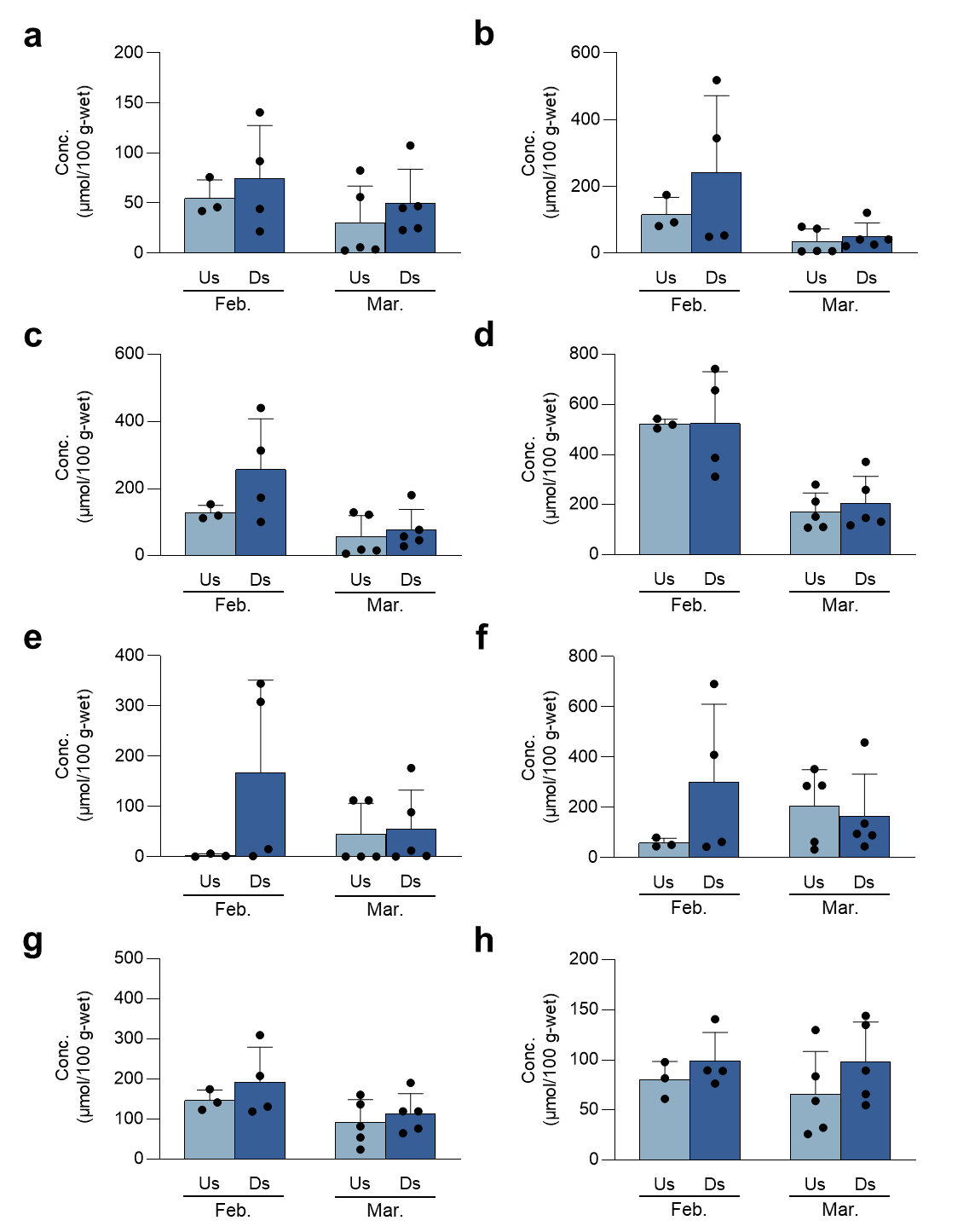


**FIGURE S8-2** Amino acid concentrations in the extracts of *T*. *osawai* collected from the up- and downstream regions of the Arakawa River (mean ± SD [µmol/100 g-wet]). Horizontal axis: sampling sites, with Us: upstream and Ds: downstream, and the month of sample collection in 2023. The concentration in each organism is indicated by a dot. L-Val (a), L-Met (b), L-Ile (c), L-Leu (d), L-Trp (e), L-Phe (f), L-Lys (g), and L-Orn (h).


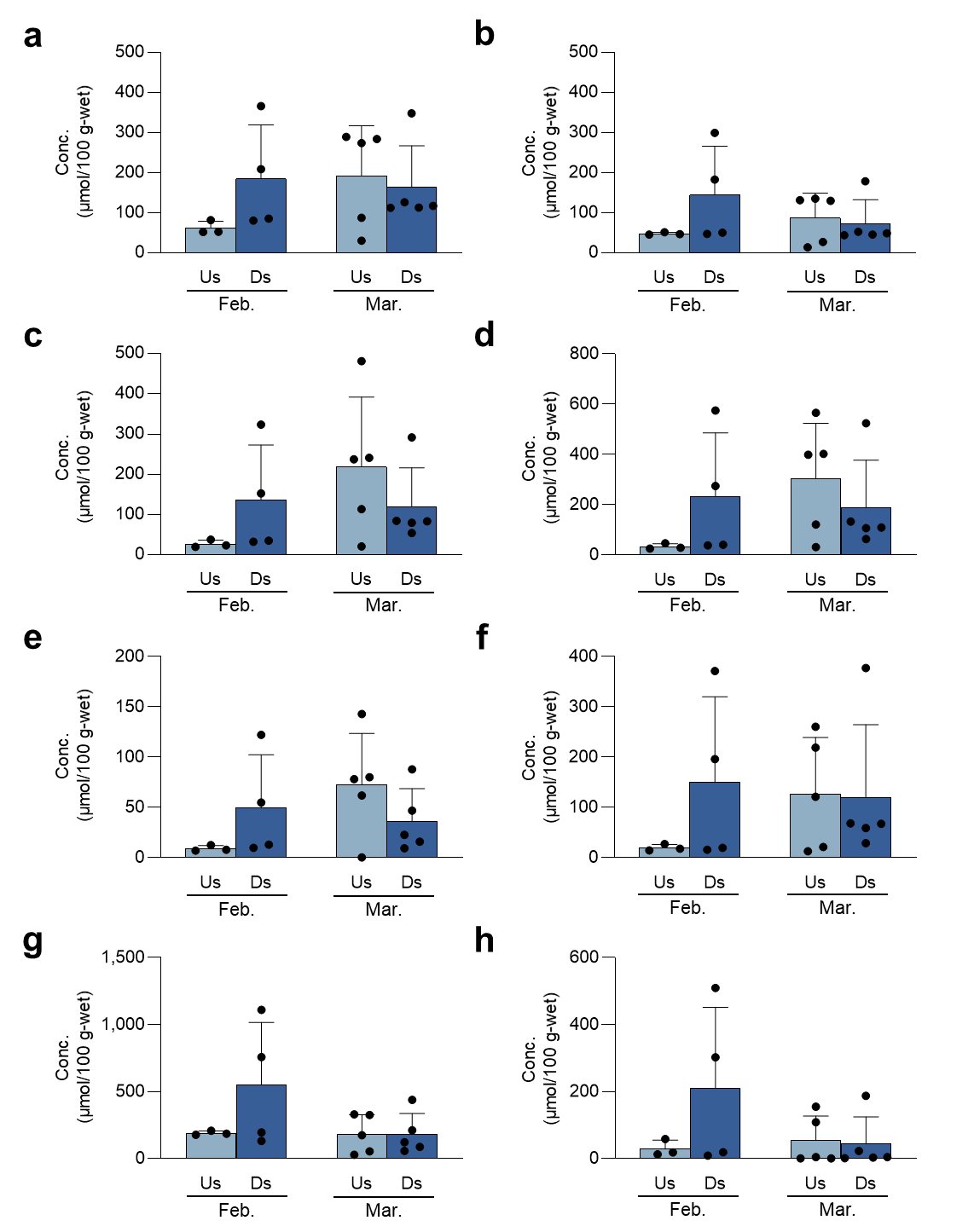


(n)

(l)

(k)

(m)

(o)

(p)

**6.9**

**7. Supporting Tables**

**FIGURE S9** Biosynthetic pathways of amino acids utilized as osmolytes in aquatic invertebrates. 3-PG: 3-phosphoglyceric acid, Enzyme 1: alanine aminotransferase, Enzyme 2: glutamic acid dehydrogenase, Enzyme 3: serine racemase, Enzyme 4: alanine racemase, Enzyme 5: aspartate racemase, and Enzyme 6: proline racemase [4,5].


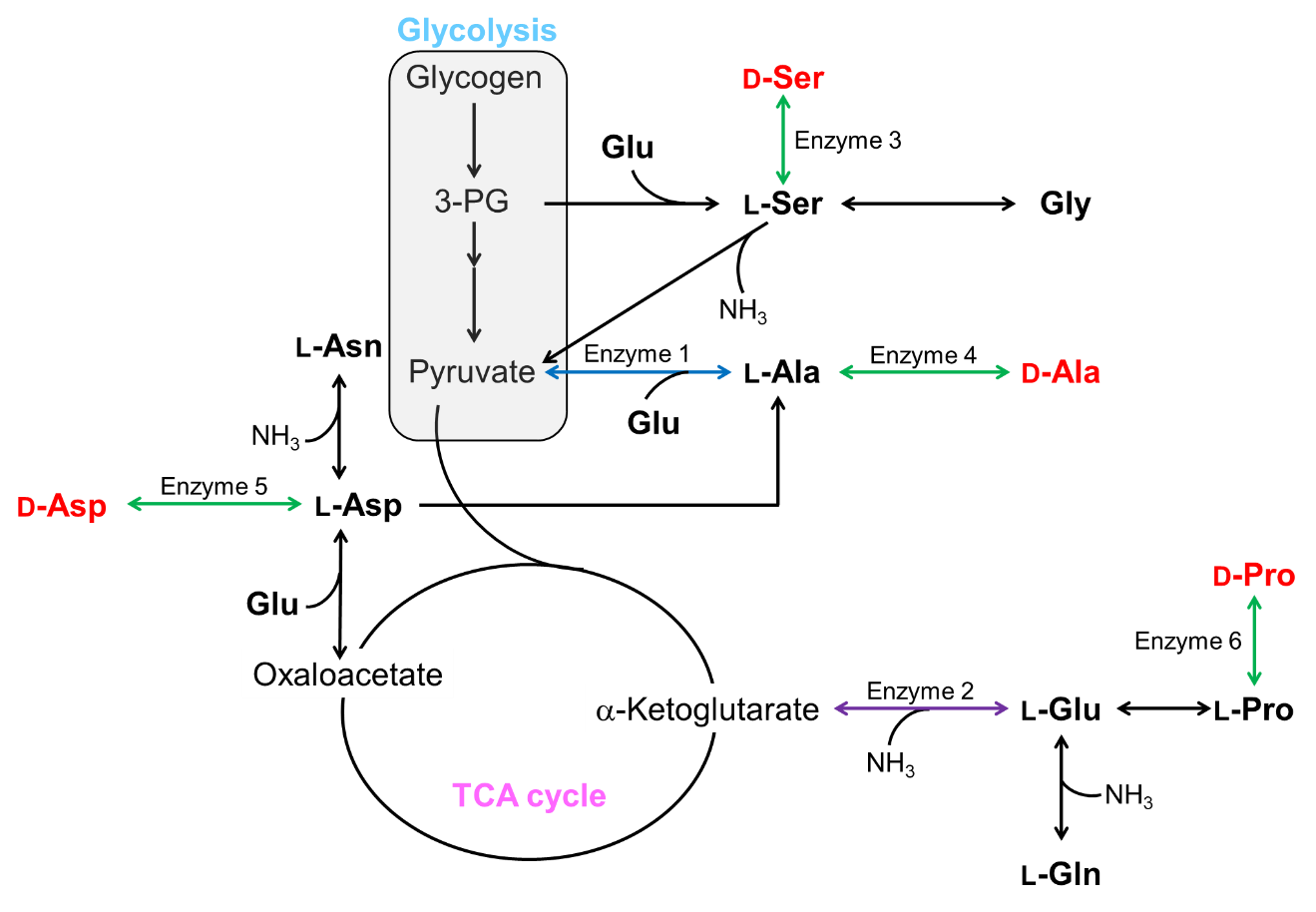


**7.1**

**Table S1** Number of polychaetes collected from each site.

**7.2**

**Table S2** Biological characteristics of polychaetes investigated in this study.

Many aspects regarding biological and ecological characteristics of polychaetes remain unclear,

and the information in this table is not exhaustive.

**7.3**

**Table S3** Results of similarity of percentages (SIMPER) analysis.

**7.4**

The population is defined as the number of individuals that may be collected by digging up the sediment.

The burrows where *T. osawai* live exhibit two types of holes: those that are currently occupied and those that were previously occupied. The latter type is so solid that it even persists during the tidal ebb and flow. Several *T. osawai* live deeper in their burrows, and several escaped during collection. Owing to these factors, the number of holes does not accurately represent the number of *T. osawai* present, and the number of holes is not equal to the population. Nevertheless, the approximate biomass may be estimated using the amount of biomass, which is determined by multiplying the mass (mean) and number of holes.

**Table S4** Biomass estimation of *T. osawai* in the Arakawa River.

**References**

[1] Sakamoto, T. *et al.* Development of derivatization reagents bearing chiral 4-imidazolidinone for distinguishing primary amines from other amino acids and application to the liquid chromatography-tandem mass spectrometric analysis of miso. *J. Chromatogr. A* **1652**, 462341; 10.1016/j.chroma.2021.462341 (2021).

[2] Onozato, M., Nakanoue, H., Sakamoto, T., Umino, M. & Fukushima, T. Determination of D- and L-amino acids in garlic foodstuffs by liquid chromatography-tandem mass spectrometry. *Molecules* **28**, 1773; 10.3390/molecules28041773 (2023).

[3] Onozato, M. *et al.* Serum d- and l-lactate, pyruvate and glucose levels in individuals with at-risk mental state and correlations with clinical symptoms. *Early Interv. Psychiatry* **14**, 410–417 (2020).

[4] Abe, H. Distribution, metabolism and physiological functions of free d-amino acids in aquatic invertebrates (in Japanese). *Vitamins* **79**, 79–86 (2005).

[5] Abe, H. Distribution of free d-amino acids and their biosyntheses and physiological roles in aquatic animals (in Japanese). *Seikagaku* **80**, 308–315 (2008).

[6] Hanafiah, Z., Sato, M., Nakashima, H. & Tosuji, H. Reproductive swarming of sympatric nereidid polychaetes in an estuary of the Omuta-gawa river in Kyushu, Japan, with special reference to simultaneous swarming of two *Hediste* species. *Zool. Sci*. **23**, 205–217 (2006).

[7] Chen, X. *et al*. First genetic assessment of brackish water polychaete *Tylorrhynchus heterochaetus*: mitochondrial COI sequences reveal strong genetic differentiation and population expansion in samples collected from southeast China and North Vietnam. *Zool. Res.* **41**, 61–69 (2020).

[8] Kasahara, S., Tokoshima, S. & Nakamura, N. An ecological study on the swarming of the so-called Japanese palolo in Ashida River. *J. Fac. Fish. Anim. Husb. Hiroshima Univ.* **11**, 79–89 (1972) (in Japanese with English abstract).

[9] Gilbert, F. *et al.* Sediment reworking by the burrowing polychaete *Hediste diversicolor* modulated by environmental and biological factors across the temperate North Atlantic. A tribute to Gaston Desrosiers. *J. Exp. Mar. Biol. Ecol.* **541**, 151588; 10.1016/j.jembe.2021.151588 (2021).

[10] Kan, K., Kuroki, Y., Sato, M., & Tosuji, H. Larval recruitment process in the catadromous life history of *Hediste diadroma* (Nereididae, Annelida) in an estuary in Kagoshima Bay, Southern Japan. *Plankton Benthos Res.* **15**, 30–43 (2020).

[11] Sato, M. & Sattmann, H. Extirpation of *Hediste japonica* (Izuka, 1908) (Nereididae, Polychaeta) in central Japan, evidenced by a museum historical collection. *Zool. Sci.* **26**, 369–372 (2009).

[12] Costa, C., Pierce, S. & Warren, M. The intracellular mechanism of salinity tolerance in polychaetes: volume regulation by isolated *Glycera dibranchiata* red coelomocytes. *Biol. Bull.* **159**, 626–638 (1980).

[13] Klawe, W. & Dickie, L. Biology of the bloodworm, *Glycera dibranchiate* Ehlera, and its relation to the bloodworm fishery of the Maritime Provinces. *Bull. Fish. Res. Bd. Canada.* **115**, 1–37 (1957).

[14] Abe, H., Tanaka, M., Taru, M., Abe, S. & Nishigaki, A. Molecular evidence for the existence of five cryptic species within the Japanese species of *Marphysa* (Annelida: Eunicidae) known as “Iwa-mushi”. *Plankton Benthos Res.* **14**, 303–314 (2019).

[15] Messina, P., Filippo, M., Gambi, M. & Zupo, V. *In vitro* fertilisation and larval development of a population of *Lumbrineris* (*Scoletoma*) *impatiens* (Claparède) (Polychaeta, Lumbrineridae) of the Gulf of Naples (Italy) in relation to aquaculture. *Inve. Repro. Develop.* **48**, 31–40 (2005).

[16] Kanaya, G., Suzuki, T. & Kikuchi, E. Spatio-temporal variations in macrozoobenthic assemblage structures in a river-affected lagoon (Idoura Lagoon, Sendai Bay, Japan): Influences of freshwater inflow. *Estu. Coastal Shelf Sci.* **92**, 169–179 (2011).

[17] Kristensen, E. *et al.* What is bioturbation? The need for a precise definition for fauna in aquatic sciences. *Mar. Ecol. Prog. Ser.* **446**, 285–302 (2012).

[18] Strathmann, R. Form, function, and embryonic migration in large gelatinous egg masses of arenicolid worms. *Invert. Biol*. **119**, 319–328 (2000).

[19] Kanaya, G. *et al.* Effects of the 2011 tsunami on the topography, vegetation, and microbenthic fauna in Gamo Lagoon, Japan. *Jap. J. Benthol.* **67**, 20–32 (2012).

[20] Kanaya, G., Suzuki, T. & Kikuchi, E. Spatio-temporal variations in macrozoobenthic assemblage structures in a river-affected lagoon (Idoura Lagoon, Sendai Bay, Japan): Influences of freshwater inflow. Estu. *Coastal Shelf Sci.* **92**, 169–179 (2013) (in Japanese with English abstract).

[21] Nishi, E. & Tanaka, K. Polychaetous annelids in tidal flats and estuaries near Kanagawa Prefecture. *Nat. History Rep. Kanagawa.* **28**, 101–107 (2007) (in Japanese with English abstract).

[22] Shaffer, P. Population ecology of *Heteromastus filiformis* (Polychaeta: Capitellidae). *Nether. J. Sea. Res.* **17**, 106–125 (1983).

[23] Wada, M., Wu, S., Kogure, K. & Tsutsumi, H. Short-term impact of biological activities of a burrowing polychaete, *Capitella* sp. I, on bacterial abundance and the chemical characteristics in organically enriched sediment. *Benth. Res.* **60**, 59–66 (2005).

[24] Blake, J. Larval development of polychaeta from the northern california coast v. *Ramex californiensis* hartman (polychaeta terebellidae). *Bull. Mar. Sci.* **48**, 448–460 (1991).

[25] Garraffoni, A. & Lana, P. Phylogenetic relationships within the Terebellidae (Polychaeta: Terebellida) based on morphological characters. *Invert. Systema.* **22**, 605–626 (2008).

[26] Garraffoni, A., Yokoyama, L. & Amaral, A. The gametogenic cycle and life history of *Nicolea uspiana* (Polychaeta: Terebellidae) on the south-east coast of Brazil. *J. Mar. Biol. Ass. U.K.* **94**, 925–933 (2014).

[27] García-Garza, M. E. & De León-González, J. A. Review of the Capitellidae (Annelida, Polychaeta) from the Eastern Tropical Pacific region, with notes on selected species. *Zookeys*. **15**, 7-52 (2011).
